# Spatial information transfer in hippocampal place cells depends on trial-to-trial variability, symmetry of place-field firing, and biophysical heterogeneities

**DOI:** 10.1016/j.neunet.2021.07.026

**Published:** 2021-07-29

**Authors:** Ankit Roy, Rishikesh Narayanan

**Affiliations:** a Cellular Neurophysiology Laboratory, Molecular Biophysics Unit, Indian Institute of Science, Bangalore, India; b Undergraduate program, Indian Institute of Science, Bangalore, India

**Keywords:** Degeneracy, Ion channels, Mutual information, Stimulus specific information, Tuning curve

## Abstract

The relationship between the feature-tuning curve and information transfer profile of individual neurons provides vital insights about neural encoding. However, the relationship between the spatial tuning curve and spatial information transfer of hippocampal place cells remains unexplored. Here, employing a stochastic search procedure spanning thousands of models, we arrived at 127 conductance-based place-cell models that exhibited signature electrophysiological characteristics and sharp spatial tuning, with parametric values that exhibited neither clustering nor strong pairwise correlations. We introduced trial-to-trial variability in responses and computed model tuning curves and information transfer profiles, using stimulus-specific (SSI) and mutual (MI) information metrics, across locations within the place field. We found spatial information transfer to be heterogeneous across models, but to reduce consistently with increasing levels of variability. Importantly, whereas reliable low-variability responses implied that maximal information transfer occurred at high-slope regions of the tuning curve, increase in variability resulted in maximal transfer occurring at the peak-firing location in a subset of models. Moreover, experience-dependent asymmetry in place-field firing introduced asymmetries in the information transfer computed through MI, but not SSI, and the impact of activity-dependent variability on information transfer was minimal compared to activity-independent variability. We unveiled ion-channel degeneracy in the regulation of spatial information transfer, and demonstrated critical roles for *N*-methyl-d-aspartate receptors, transient potassium and dendritic sodium channels in regulating information transfer. Our results demonstrate that trial-to-trial variability, tuning-curve shape and biological heterogeneities critically regulate the relationship between the spatial tuning curve and spatial information transfer in hippocampal place cells.

## 1 Introduction

Biological organisms rely on information about their surroundings through different senses for survival. They receive, encode and process information about their surroundings in eliciting robust responses to challenges posed by the external environment. From an ethological perspective, it is essential that sensory information is efficiently encoded by neural circuits to ensure effective responses to environmental challenges. A dominant theme of neural circuit organization is the ability of individual neurons to encode specific features associated with the external environment, with different neurons responding maximally to distinct feature values. For instance, neurons in the primary visual cortex respond maximally to a specific visual orientation (Hubel & Wiesel, 1959), neurons in the cochlea respond maximally to specific tones (von Békésy & Wever, 1960) and place cells in the hippocampus act as spatial sensors by responding maximally to specific locations of an animal in its environment (O’Keefe, 1976). Central to this overarching design principle is the concept of tuning curves, whereby neurons that respond maximally to a given feature value also respond to nearby feature values, with the response intensity typically falling sharply with increasing feature distance from the peak-response feature. The concept of “tuning curves” and efficient information transfer involving stimulus distributions have been effectively employed to assess biological systems from the sensory coding perspective (Attneave, 1954; Barlow, 1961; Bell & Sejnowski, 1997; Brenner, Bialek, & de Ruyter van Steveninck, 2000; Fairhall, Lewen, Bialek, & de Ruyter Van Steveninck, 2001; Laughlin, 1981; Lewicki, 2002; Simoncelli, 2003; Simoncelli & Olshausen, 2001), from a single neuron perspective (Andrews & Iglesias, 2007; Lundstrom, Higgs, Spain, & Fairhall, 2008; Narayanan & Johnston, 2012; Stemmler & Koch, 1999) and in understanding biochemical signaling cascades (Brennan, Cheong, & Levchenko, 2012; Cheong, Rhee, Wang, Nemenman, & Levchenko, 2011; Mehta, Goyal, Long, Bassler, & Wingreen, 2009; Selimkhanov et al., 2014; Tkacik, Callan, & Bialek, 2008; Waltermann & Klipp, 2011; Yu et al., 2008).

A fundamental question on neurons endowed with such tuning curves relates to the relationship between the tuning curve and the information transfer profile of the neuron across feature values. Although this relationship has been explored in neural responses across different sensory modalities (Bezzi, Samengo, Leutgeb, & Mizumori, 2002; Butts, 2003; Butts & Goldman, 2006; DeWeese & Meister, 1999; Montgomery & Wehr, 2010), the question on the relationship between spatial information transfer and spatial tuning curve *within* the place field of hippocampal place cells has not been quantitatively assessed. Neurons in the hippocampus receive spatial information about a given arena and a substantial fraction of them respond to different spatial locations in the same arena (Andersen, Morris, Amaral, Bliss, & O’Keefe, 2006; Moser, Kropff, & Moser, 2008; Moser, Moser, & McNaughton, 2017; Moser, Rowland, & Moser, 2015; O’Keefe, 1976; O’Keefe & Dostrovsky, 1971). In a one-dimensional arena, hippocampal place cells exhibit bell-shaped firing *within* their place-field firing, representing a tuning curve of the external space (Ahmed & Mehta, 2009; Bittner et al., 2015; Bittner, Milstein, Grienberger, Romani, & Magee, 2017; Dombeck, Harvey, Tian, Looger, & Tank, 2010; Dragoi & Buzsaki, 2006; Geisler et al., 2010; Grienberger, Milstein, Bittner, Romani, & Magee, 2017; Harvey, Collman, Dombeck, & Tank, 2009; Huxter, Burgess, & O’Keefe, 2003; Lee, Lin, & Lee, 2012; Mehta, Barnes, & McNaughton, 1997; Mehta, Lee, & Wilson, 2002; Mehta, Quirk, & Wilson, 2000). The specific question we pose here is on the relationship between this tuning curve and the spatial information transfer with reference to synaptic inputs received by the place cell (that contains spatial information from the external world) and a specific output characteristic (rate of firing). In this scenario, spatial information transfer is computed with reference to a variable associated with the external world, the spatial location *within* the place field, and the firing of the neuron. These definitions of tuning curves and information transfer are analogous to the assessment of information transfer in cortical neurons receiving sensory inputs that traverse through multiple synapses. As an example, for neurons in the visual cortex (which are several synapses away from the eyes), orientation-selective tuning curves and visual information transfer questions are posed with reference to the synaptic inputs received by the neuron (containing visual information from the external world) and a specific output characteristic (*e.g.,* spikes, rate of firing) (Belitski et al., 2008; Bell & Sejnowski, 1997; Hubel & Wiesel, 1959).

Spatial tuning curves, by definition, are dependent on specific spatial locations *within* the place field. As our principal goal in this study is to assess the relationship between spatial tuning curves and spatial information transfer, it is essential that the information transfer measure also is specific to particular spatial locations. An ideal information metric that fulfills this requirement is the stimulus-specific information (SSI), a measure that was specifically defined to convey the amount of information that the responses of a neuron convey about *a particular stimulus.* SSI is defined as the average *specific information* across all the neural firing rates that are elicited when the animal traverses a particular spatial location, with *specific information* referring to the information that a *particular* firing rate response provides about which spatial location was being traversed (Butts, 2003; Butts & Goldman, 2006; DeWeese & Meister, 1999; Montgomery & Wehr, 2010). We employed SSI as the principal metric to assess the relationship between rate-based spatial tuning curves and spatial information transfer. We also computed the Shannon’s mutual information (MI) at *different segments* within the place field as an additional location-dependent information metric. Whereas the SSI offers a weighted average of specific information, which is a metric that accounts for *all* spatial locations within the place field, the location-dependent MI that we computed solely accounts for firing rate responses within a small segment of the entire place field.

In assessing the relationship between spatial information transfer and spatial tuning curves, it was essential to account for three characteristics in our experimental design:

1. Biological neurons are heterogeneous. Neurons of the same cell type from the same subregion show very distinct ion channel distributions, even if they maintain signature electrophysiological properties. These observations pose two important questions:
  - How do neurons maintain such signature electrophysiological properties despite widespread variability in ion-channel distribution?
  - What are implications for the expression of these heterogeneities neuronal functions, including encoding of external stimulus and information transfer?
  An elegant answer to the first question is provided by noting that biological systems express *degeneracy,* defined as the ability of multiple structural components to elicit the same function (Edelman & Gally, 2001). Although CA1 pyramidal neurons have been shown to exhibit degeneracy with reference to several functional outcomes (Basak & Narayanan, 2018, 2020; Das, Rathour, & Narayanan, 2017; Jain & Narayanan, 2020; Migliore et al., 2018; Rathour, Malik, & Narayanan, 2016; Rathour & Narayanan, 2012, 2014, 2019), it is not known if distinct combinations of ion channel conductances elicit similar functional outcomes — both in terms of spatial information transfer and neuronal intrinsic properties.
  The answer to the second question on the impact of heterogeneities on neural function is critical from physiological and pathological perspectives. Experimental analyses typically interpret outcomes from summary statistics, and computational models often employ a *single hand-tuned model* to arrive at conclusions. This approach is perilous (Marder & Taylor, 2011; Rathour & Narayanan, 2019) because a single hand-tuned model *does not reflect* the biological heterogeneities, and thus provides incorrect conclusions about the contributions of individual ion channels (or other parameters) to distinct functions. Thus, we constructed a population of heterogeneous models, which were morphologically realistic and conductance-based, in arriving at our interpretations and conclusions. This heterogeneous conductance-based modeling framework allowed us to effectively address the second question on the impact of biological heterogeneities on spatial information transfer, and to assess the impact of individual ion channels and receptors on spatial information transfer.
2. The relationship between tuning curves and information transfer depends on trial-to-trial variability in other neural systems (Butts & Goldman, 2006; Montgomery & Wehr, 2010). Specifically, it has been shown that the maximal information transfer occurs at high-slope regions under low variability, and switches to the peak-firing region of the tuning curve when variability increases. To address this question in our heterogeneous population of CA1 place cells, we subjected this population to different levels of activity-independent or activity-dependent trial-to-trial variability, and assessed the relationship between spatial information transfer and spatial tuning curves.
3. Hippocampal place cells manifest experience-dependent asymmetry in their place-field firing (Harvey et al., 2009; Mehta et al., 1997, 2002, 2000). We utilized these observations to analyze the impact of experience-dependent asymmetry on spatial tuning curves, spatial information transfer, and the relationship between the two measurements.

Our analyses show that each of these three characteristics — biophysical and physiological heterogeneities, the type and level of trial-to-trial variability, and behavior-dependent alterations to the tuning curve — critically regulated the relationship between the spatial tuning curve and spatial information transfer. We demonstrate that when hippocampal neurons exhibit low trial-to-trial response variability, they transfer peak spatial information at the high-slope locations (and not at peak firing location) of the spatial tuning curve within their place field. Importantly, we show that our model population manifested parametric degeneracy in the expression of similar tuning curves and similar information transfer metrics. As a consequence of the expression of degeneracy, we found heterogeneities in spatial information transfer and in the impact of knocking out individual ion channels on spatial information metrics, together pointing to a many-to-one relationship between different ion channel subtypes and spatial information transfer. Finally, our analyses also unveil a potent reduction in information transfer consequent to the elimination of transient potassium channels, NMDA receptors or dendritic sodium channels, thereby providing direct experimentally testable predictions.

## 2 Methods

**Fig. 1:**
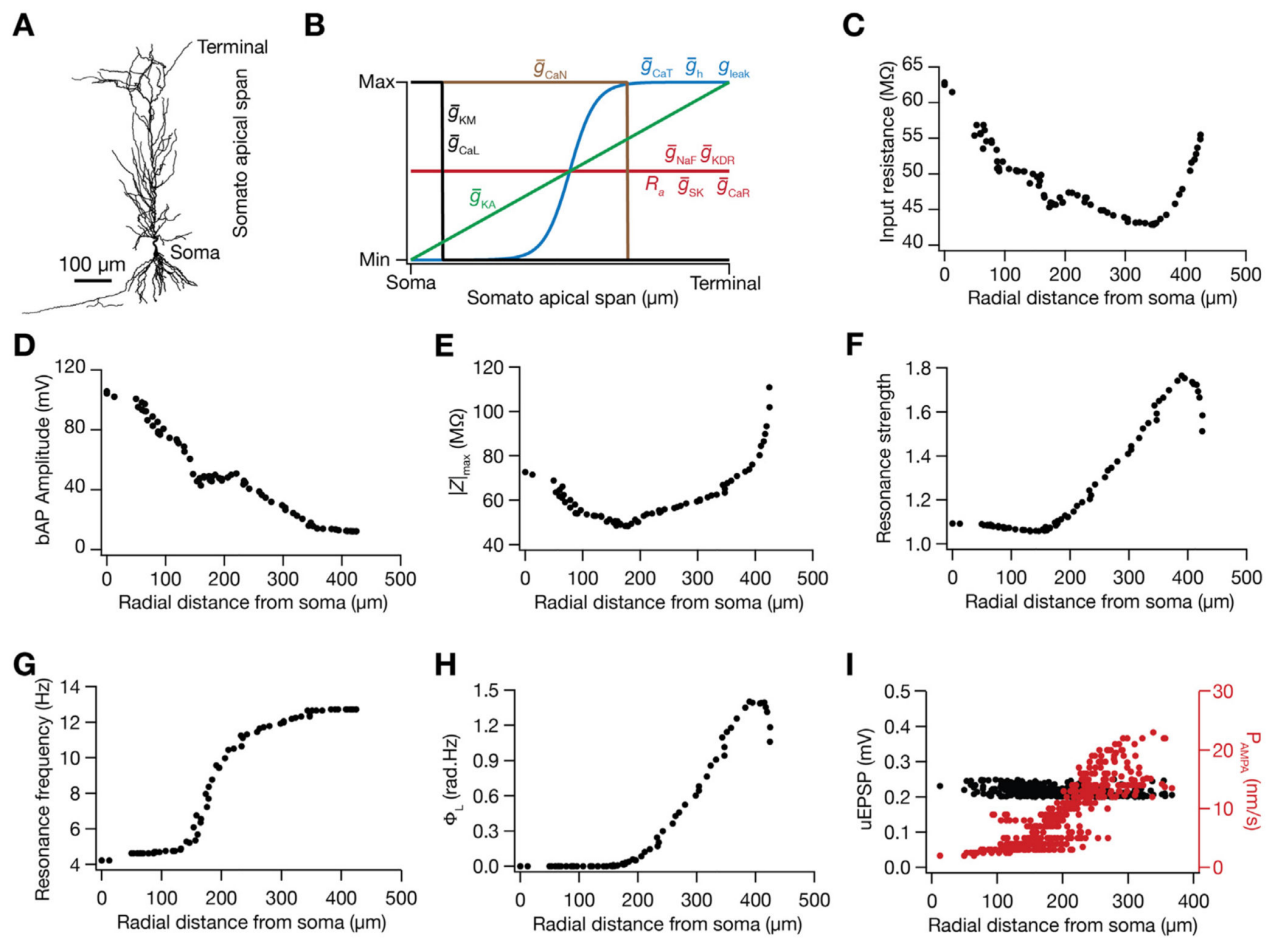
Base model of rat hippocampal CA1 pyramidal neurons, showing its intrinsic and synaptic properties along the somato-apical trunk. (A) Two-dimensional reconstruction of the 3D morphologically realistic model employed in this study. (B) Distribution of parameters governing the passive properties *g_leak_* and *R_a_*) and ten different active ion channels (*g_h_*, *g_NaF_*, *g_KDR_*, *g_KA_*, *g_KM_*, *g_SK_*, *g_CaN_*, *g_CaL_*, *g_CaR_* and *g_CaT_*) along the somato-apical span to match multiple intrinsic measurements at the soma and along the apical dendrites, including input resistance (C), backpropagating action potential amplitude (D) maximum impedance amplitude (E), strength of resonance (F) resonance frequency (G), total inductive phase (H) and the maximum AMPAR permeability (I), all as functions of radial distance from the soma. The distance-dependent profile of maximum AMPAR permeability, *P*_AMPA_ (I, right vertical axis) was set such that the somatic unitary excitatory postsynaptic potentials (uEPSPs) were around 0.2 mV, irrespective of synaptic location (*I*, left vertical axis).

The computational model of the place cell was constructed as a morphologically realistic CA1 pyramidal neuron of rat hippocampus. A morphologically reconstructed model (n123; Fig. 1A) was obtained from Neuromorpho.org (Ascoli, Donohue, & Halavi, 2007). Several active and passive mechanisms were incorporated into the model to mimic intrinsic functional properties of a CA1 pyramidal neuron. The passive properties arising due to the lipid bilayer was modeled as a capacitive current, and to represent the leak channels a resistive current was included. The three parameters which regulated the passive electrical properties of the neuron are axial resistivity (*R_a_*), specific membrane resistivity (*R_m_*) and specific membrane capacitance (*C_m_*). In the base model, *R_a_* was set to 120 Ωcm and the specific membrane capacitance was set to 1 μF/cm^2^ for the entire neuron (Table 1, Fig. 1B). The specific membrane resistivity was non-uniform and varied in a sigmoidal manner (Basak & Narayanan, 2018; Golding, Mickus, Katz, Kath, & Spruston, 2005; Narayanan & Johnston, 2007; Rathour & Narayanan, 2014) as a function of the distance of the point from the soma (*x*) (Fig. 1B):

(1) $$R_{m}(x)=R_{m}^{\text{soma}\;}+\frac{R_{m}^{\text{end}\;}-R_{m}^{\text{soma}\;}}{1+\text{exp}\left(R_{m}^{\text{hmp}}-x\right)/R_{m}^{\text{slope}\;}}$$

In Eq. (1), *x* is the radial distance from soma, and the parameters and their base-model values are provided in Table 1. The neuron was compartmentalized using the *d_λ_* rule (Carnevale & Hines, 2006), such that the length of each compartment was less than one-tenth of λ_100_, the space constant at 100 Hz. In the base model, this resulted in the compartmentalization of the neuron into 879 distinct compartments.

To model the active properties of the neuron, 10 different types of ion channels were incorporated into the base model, based on electrophysiological characterization from CA1 pyramidal neurons. The ion channels incorporated were the fast sodium (NaF), delayed rectifier potassium (KDR), *A*-type potassium (KA), *M*-type potassium (KM), small-conductance calcium activated potassium (SK), *T*-type calcium (CaT), *N*-type calcium (CaN), *R*-type calcium (CaR), *L*-type calcium (CaL) and hyperpolarisation activated cyclic nucleotide gated (HCN or *h*). The current through these channels due to Na^+^, K^+^ ions were modeled in an ohmic formulation with the reversal potentials of Na^+^, K^+^ and *h* channels being 55, −90 and −30 mV respectively. The current due to calcium ions was modeled as per the Goldman–Hodgkin–Katz (GHK) conventions with the internal calcium concentration as 50 nM and external calcium concentration as 2 mM. The equations underlying the kinetics of these channels were obtained from prior electrophysiological recordings: NaF, KDR and KA (Hoffman, Magee, Colbert, & Johnston, 1997; Magee & Johnston, 1995; Migliore, Hoffman, Magee, & Johnston, 1999), HCN (Magee, 1998), KM (Shah, Migliore, Valencia, Cooper, & Brown, 2008), SK (Sah & Clements, 1999; Sah & Isaacson, 1995), CaT (Shah, Migliore, & Brown, 2011), CaN (Migliore, Cook, Jaffe, Turner, & Johnston, 1995), CaR and CaL (Magee & Johnston, 1995; Poirazi, Brannon, & Mel, 2003).

These ion channels were distributed along the somatodendritic axis to match experimental recordings (Table 1 provides the distributions and the parameter values in the base model). Specifically, the fast sodium and the delayed rectifier potassium were uniformly distributed (Bittner, Andrasfalvy, & Magee, 2012; Hoffman et al., 1997; Magee & Johnston, 1995). The *A*-type potassium channel density increased linearly (Hoffman et al., 1997) as a function of distance from soma, *x* (Fig. 1B):

(2) $$g_{KA}(x)={\bar{g}}_{KA}^{\text{soma}}\left(1+\frac{{\bar{g}}_{KA}^{\text{fold}}}{100}x\right)$$

The HCN and *T*-type calcium channel density were set in a sigmoidal manner (Fig. 1B), increasing with radial distance from the soma (Lorincz, Notomi, Tamas, Shigemoto, & Nusser, 2002; Magee, 1998; Magee & Johnston, 1995; Narayanan & Johnston, 2007; Rathour & Narayanan, 2014):

(3) $$g_{h}(x)={\bar{g}}_{h}^{\text{soma}\;}\left(1+\frac{{\bar{g}}_{h}^{\text{fold}\;}}{1+\text{exp}\left(\left({\bar{g}}_{h}^{\text{hmp}}-x\right)/{\bar{g}}_{h}^{\text{slope}\;}\right)}\right)$$

(4) $$g_{CaT}(x)={\bar{g}}_{CaT}^{\text{soma}\;}\left(1+\frac{{\bar{g}}_{CaT}^{\text{fold}\;}}{1+\text{exp}\left(\left({\bar{g}}_{CaT}^{\text{hmp}}-x\right)/{\bar{g}}_{CaT}^{\text{slope}\;}\right)}\right)$$

The *M*-type potassium and *L*-type calcium channels were perisomatic (Hu, Vervaeke, & Storm, 2007; Magee & Johnston, 1995). The SK and the *R*-type calcium channels were distributed uniformly across the apical dendrites (Lin, Lujan, Watanabe, Adelman, & Maylie, 2008; Magee & Johnston, 1995; Ngo-Anh et al., 2005). The *N*-type calcium channels were uniformly distributed till 340 μm of radial distance along the apical dendrite (Magee & Johnston, 1995). The distances are specified as radial distances to match with experimental measurements that are conventionally reported as radial distances, and not path distances from the soma (*e.g.,* Bittner et al., 2012; Hoffman et al., 1997; Magee, 1998; Magee & Johnston, 1995; Narayanan & Johnston, 2007; Spruston, Schiller, Stuart, & Sakmann, 1995).

### 2.1 Intrinsic physiological measurements

To measure input resistance (*R*_in_) of a somatodendritic compartment, a hyperpolarizing current step of 100 pA was injected for 500 ms into the compartment. The local change in the membrane potential as a result of the step current was measured and the ratio of the local voltage deflection to the step current amplitude was taken to be the input resistance (Fig. 1C). For measuring the back propagating action potential (bAP) amplitude, a step current of 1 nA was given at the soma for 2 ms. This generated a single action potential at the soma which actively back propagated along the dendrites. The amplitude of the bAP was measured at different locations along the somato-apical trunk (Fig. 1D).

To quantify the frequency dependence of neuronal responses, we used impedance based physiological measurements across the somatodendritic arbor Basak and Narayanan (2018, 2020), Narayanan, Dougherty, and Johnston (2010), Narayanan and Johnston (2007, 2008) and Rathour and Narayanan (2014): resonance frequency (*f*_R_), maximum impedance amplitude (|*Z*|_max_), strength of resonance (*Q*) and total inductive phase (Φ_L_). To measure these a chirp stimulus, defined as a current stimulus with constant amplitude (peak to peak 100 pA) and linearly increasing frequency with time (0–15 Hz in 15 s), was injected in the compartment where the measurement was required. The local voltage response was recorded. To compute the impedance as a function of frequency, the Fourier spectrum of voltage response was divided with the Fourier spectrum of the current giving us the impedance profile as a complex quantity. The magnitude of impedance as a function of frequency was calculated using the following equation,

(5) |Z(f)|=Re(Z(f))2+Im(Z(f))2

In Eq. (5), Re (*Z* (*f*)) is the real part of the impedance profile and Im (*Z* (*f*)) is the imaginary part of the impedance profile and |*Z* (*f*) | is the magnitude of impedance. The maximum impedance amplitude was measured and the frequency at which it occurred was taken to be the resonance frequency. The strength of resonance was measured by taking ratio of the maximum impedance amplitude to the impedance amplitude at 0.5 Hz. For the phase related measures, the impedance phase profile was computed:

(6) ϕ(f)=tan−1Im(Z(f))Re(Z(f))

In Eq. (6), *ϕ (f*) is the phase as a function of frequency. The total inductive phase was measured by calculating the area under the positive portion of phase profile:

(7) $${\text{Φ}}_{\text{L}}=\int_{\phi (f)>0}\phi (f)df$$

### 2.2 Synapses and normalization of somatic unitary synaptic potential

The model contained excitatory synapses with colocalized NMDAR and AMPAR, with an NMDAR-to-AMPAR ratio of 1.5, with 80 such synapses randomly dispersed across the apical dendritic arbor (Basak & Narayanan, 2018, 2020). These 80 synapses correspond to the number of *active* synapses when the animal traverses *within* the place field of the postsynaptic neuron. The number of synapses was based on sensitivity analyses spanning different synapse numbers (Basak & Narayanan, 2018). Broadly, neural firing rate was directly related to the number of synapses, but resulted in depolarization-induced block if number of synapses increased beyond a certain threshold (Basak & Narayanan, 2018). The current through the NMDAR were divided into current due to three ions, Na^+^, K^+^ and Ca^2+^. The dependence of current due to each of these ions as a function of voltage and time was modeled in GHK formulation (Anirudhan & Narayanan, 2015; Ashhad & Narayanan, 2013; Basak & Narayanan, 2018, 2020; Narayanan & Johnston, 2010):

(8) $$I_{NMDA}(v,t)=I_{NMDA}^{Na}(v,t)+I_{NMDA}^{K}(v,t)+I_{NMDA}^{Ca}(v,t)$$

(9) $$\begin{array}{c} I_{NMDA}^{Na}(v,t)={\bar{P}}_{NMDAR}P_{Na}S\;(t)\text{MgB}\;(v)\frac{vF^{2}}{RT}\\ \times \left(\frac{{\left[Na\right]}_{i}-{\left[Na\right]}_{o}\text{exp}\left(-\frac{vF}{RT}\right)}{1-\text{exp}\left(-\frac{vF}{RT}\right)}\right) \end{array}$$

(10) $$\begin{array}{c} {\text{I}}_{NMDA}^{K}(\text{v},\text{t})={\bar{P}}_{NMDAR}P_{K}s\;(\text{t})\text{MgB}\;(v)\frac{v{\text{F}}^{2}}{RT}\\ \times \left(\frac{{\left[K\right]}_{i}-{\left[K\right]}_{0}\text{exp}\left(-\frac{vF}{RT}\right)}{1-\text{exp}\left(-\frac{vF}{RT}\right)}\right) \end{array}$$

(11) $$\begin{array}{c} I_{NMDA}^{Ca}(v,t)={\bar{P}}_{NMDAR}P_{Ca}S(t)MgB(v)\frac{4vF^{2}}{RT}\\ \times \left(\frac{{\left[\text{Ca}\right]}_{i}-{\left[\text{Ca}\right]}_{o}\text{exp}\left(-\frac{2vF}{RT}\right)}{1-\text{exp}\left(-\frac{2vF}{RT}\right)}\right) \end{array}$$

Here, ${\bar{P}}_{NMDAR}$ defined the maximum permeability of NMDA receptors. The relative permeability ratios were set to *P_Ca_ =* 10.6, *P_Na_ =* 1 and *P*_K_ = 1. The ionic concentrations were set as, [*Na*]_*i*_ = 18 mM, [*Na*]_*o*_ = 140 mM, [*K*]_*i*_ = 140 mM, [*K*]_*o*_ = 5 mM, [*Ca*]_*i*_ = 100 nM and [*Ca*]_*o*_ = 2 mM. The magnesium dependence of the NMDAR current was calculated as follows (Jahr & Stevens, 1990):

(12) $$\text{MgB}\;(v)={\left(1+\frac{{\left[Mg\right]}_{0}\text{exp}(-0.062v)}{3.57}\right)}^{-1}$$

with [*Mg*]_*o*_ = 2 mM. The kinetics of the NMDAR current was determined by *s (t*):

(13) $$s(t)=a\left(\text{exp}\left(-\frac{t}{\tau_{d}}\right)-\text{exp}\left(-\frac{t}{\tau_{r}}\right)\right)$$

Here *a* is a normalization constant such that 0 ≤ *s (t*) ≤ 1, *τ_d_* is the decay constant, *τ_r_* is the rise time, with *τ_r_ =* 5 ms and default *τ_d_ =* 50 ms (Ashhad & Narayanan, 2013; Narayanan & Johnston, 2010).

The current through the AMPA receptor was mediated by two ions, Na^+^ and K^+^.

(14) $$I_{AMPA}(v,t)=I_{AMPA}^{Na}(v,t)+I_{AMPA}^{K}(v,t)$$

(15) $$I_{AMPA}^{Na}(v,t)={\bar{P}}_{AMPAR}P_{Na}S(t)\frac{vF^{2}}{RT}\left(\frac{{\left[Na\right]}_{i}-{\left[Na\right]}_{o}\text{exp}\left(-\frac{vF}{RT}\right)}{1-\text{exp}\left(-\frac{vF}{RT}\right)}\right)$$

(16) $$I_{AMPA}^{K}(v,t)={\bar{P}}_{AMPAR}P_{K}S(t)\frac{vF^{2}}{RT}\left(\frac{{\left[K\right]}_{i}-{\left[K\right]}_{o}\text{exp}\left(-\frac{vF}{RT}\right)}{1-\text{exp}\left(-\frac{vF}{RT}\right)}\right)$$

In Eqs. (15)–(16), *P̄_AMPAR_*, defined the maximum permeability of AMPA receptors. The relative permeability ratios were set to *P_Na_ =* 1 and *P_k_ =* 1. The *s (t*) was modeled in a manner similar to NMDAR with *τ_r_ =* 2 ms and *τ_d_ =* 10 ms. To normalize the unitary EPSP values associated with each synapse, we ensured that the attenuation along the dendritic cable did not affect the unitary somatic EPSP amplitude. Hence, the AMPAR permeabilities at the somato-apical trunk was tuned such that it produced a unitary somatic response of ~ 0.2 mV irrespective of the synaptic location (Andrasfalvy & Magee, 2001; Magee & Cook, 2000).

### 2.3 Place cell inputs and synaptic localization

The input to this neuron was fed through colocalized AMPAR-NMDAR synapses. As the virtual animal traversed through the place field the presynaptic neurons fired action potentials. Their firing rates were modeled in a stochastic manner, driven by a Gaussian modulated cosinusoidal function, mimicking place cell inputs to the neuron (Basak & Narayanan, 2018, 2020; Seenivasan & Narayanan, 2020). The presynaptic firing drove the opening of the colocalized synaptic NMDAR and AMPARs, resulting in synaptic currents (Eqs. (8)–(16)) flowing into the model neuron. The Gaussian modulated cosinusoidal function that governed the probability of occurrence of a presynaptic spike to each synapse in the neuron was computed as (Basak & Narayanan, 2018, 2020; Seenivasan & Narayanan, 2020):

(17) $$F_{\text{pre}}(t)=F_{\text{pre}}^{\max}\left(1+\cos\left(2\pi f_{0}(t-T)\right)\right)\text{exp}\left(-\frac{{\left(t-T\right)}^{2}}{2\sigma^{2}}\right)$$

In Eq. (17), *T* (5 s) defined the center of the place field, *f*_0_ is the frequency of the cosine (8 Hz), $F_{\text{pre}}^{\text{max}}$ is the maximal input firing rate, *σ* is the standard deviation of the Gaussian (1 s). In our analyses, the virtual animal was assumed to traverse a linear arena at constant velocity, implying the equivalence of time and space as the independent variable in Eq. (17). The input current resulting from synaptic activation produced post-synaptic action potentials and caused place cell like firing activities in the model neuron.

In introducing experience-dependent asymmetry in place-field firing (Harvey et al., 2009; Mehta et al., 1997, 2002, 2000), we replaced the symmetric Gaussian profile in Eq. (17) by a horizontally reflected Erlang distribution to construct an asymmetric place-field envelope (Seenivasan & Narayanan, 2020). In this scenario, the Erlang-modulated cosinusoidal function that governed the probability of occurrence of a presynaptic spike to each synapse in the neuron was computed as:

(18) $$F_{\text{pre}\;}(t)=F_{\text{pre}\;}^{\max}\left(1+\cos\left(2\pi f_{0}(t-T)\right)\right)\frac{\lambda^{k}{\left(T-t\right)}^{k-1}e^{-\lambda (T-t)}}{(k-1)!}$$

In Eq. (18), the parameters λ (=5) and *k* (=25) governed the extent of asymmetry (Seenivasan & Narayanan, 2020).

Although each of the 80 synapses was driven by the Gaussian- or the Erlang-modulated cosinusoidal functions for the probabilistic generation of their respective pre-synaptic spike trains, they were independently generated, thereby ensuring that the input spikes are not temporally synchronous. Specifically, for a given synapse, at each integration time step (*dt* = 25 μs), a random number was generated from a uniform random distribution spanning (0,1). An event corresponding to a presynaptic spike for this synapse was generated if this random number was less than *dt* × *F_pre_(t*) at a given time *t*. This process was *independently* repeated for each *dt* across each of the 80 synapses impinging on the postsynaptic neuron.

### 2.4 Trial-to-trial variability in place-cell responses

For simulating trial-to-trial variability in the place cell firing profile with different levels of variability, noise was introduced into the presynaptic firing rate profile (Eq. (17)) associated with each synapse. Simulations were performed with Gaussian white noise (GWN) which was introduced either additively (AGWN) or multiplicatively (MGWN):

(19) $$F_{\text{pre}\;}^{\text{AGWN}\;}(t)={\left[F_{\text{pre}\;}(t)+\xi (t)\right]}^{+}$$

(20) $$F_{\text{pre}}^{\text{MGWN}}(t)={\left[F_{\text{pre}}(t)(1+\xi (t))\right]}^{+}$$

In Eqs. (19)–(20), [*F*]^+^ = max(*F*, 0) represents rectification to avoid negative firing rates, ξ(*t*) defined a GWN with zero mean and standard deviation σ_noise_. As the rectification governs the overall firing rate and not the noise term, this formulation allows for both negative and positive modulation of *F*_pre_ (*t*). The value of σ_noise_ was increased to enhance the level of trial-to-trial variability, with *F*_pre_ (*t*) defined by a Gaussian- (Eq. (17)) or an Erlangenvelope (Eq. (18)) to assess the impact of trial-to-trial variability in symmetric or asymmetric place-field firing profiles, respectively. As AGWN (Eq. (19)) introduced trial-to-trial variability across stimulus locations, irrespective of the strength of afferent synaptic activity, this form of variability is *activity-independent*. On the other hand, the level of trial-to-trial variability introduced by MGWN is progressively higher with increasing strength of afferent synaptic activity (Eq. (20)), thereby manifesting as *activity-dependent* trial-to-trial variability.

### 2.5 Neuronal voltage response during place-field traversal

Spikes were detected from the place-cell voltage response to afferent synaptic stimuli (Eqs. (17)–(20)) by setting a voltage threshold on the rising phase of the voltage values. These spike timings were then converted to the firing rate of the place cell as a function of time (*F*(*t*)) through convolution with a Gaussian kernel (σ = 200 ms). The maxima (*F*_max_) and the full-width at half maximum (*FWHM*) of the place-cell firing profile were employed as *relative* measures of place-field tuning sharpness. Specifically, high *F*_max_ and low *FWHM* (Table 2) were indicative of a sharply tuned place-cell responses (Basak & Narayanan, 2018, 2020). We took this *relative* approach of using *high F*_max_ and *low FWHM* for assessing tuning sharpness to ensure that our comparisons of the model remain focused on synaptic and channel localization profiles. Specifically, we resorted to these *relative* metrics to circumvent heterogeneities in spatial extent of place-cell populations, especially along the dorso-ventral axis (Kjelstrup et al., 2008; Strange, Witter, Lein, & Moser, 2014). Our experimental design involves the assessment of responses of the model cell are to a Gaussian- (Eq. (17)) or Erlang-modulated (Eq. (18)) cosinusoidal waveform with a *fixed* width. With the input distribution fixed, this design allowed us to focus specifically on the roles of the neuron's intrinsic properties and of synaptic localization on the output tuning profiles and spatial information transfer (Basak & Narayanan, 2018, 2020).

As animals traverse through the place field of a given hippocampal place cell, these neurons are known to produce characteristic sub-threshold voltage ramps (Harvey et al., 2009). To assess such ramps, we filtered the voltage traces using a 0.75 s wide median filter, which removed the spikes and exposed the sub-threshold structure of the voltage response during place-field traversal. The maximum value of these ramps was taken as peak ramp voltage (*V*_ramp_). Since the firing rate of the presynaptic neurons were modulated with a sinusoid of theta frequency (8 Hz, Eqs. (17)–(18)), we analyzed whether the post synaptic voltage traces reflected this temporal modulation. The voltage trace at the soma was filtered using a 50 ms wide median filter, to eliminate spikes but retain theta-frequency temporal modulation, and the Fourier spectrum of the filtered signal was computed. The power at 8 Hz of this power spectrum represented theta power (Basak & Narayanan, 2018, 2020; Seenivasan & Narayanan, 2020).

### 2.6 Spatial information transfer within a place field: Mutual information metrics

To quantify the information transmitted through the firing pattern of a place cell, we employed two sets of information metrics. The first set involved the computation of mutual information (MI), with space within the place field considered as the stimulus and the neuronal firing-rate considered the response. The aforementioned equivalence of time and space as the independent variable in Eqs. (17)–(20) allowed us to compute spatial information transfer from the firing rate response.

To obtain location-dependent spatial information transfer, we computed mutual information in a piece-wise manner at 20 different locations (*N*loc) from the instantaneous firing-rate profile obtained for 30 different trials. To compute MI at these 20 locations, each location was subdivided into 4 bins, and the associated firing rate response was quantized into 20 bins. Mutual information between the spatial stimulus (*S*) and firing-rate response (*F*) was calculated at each *N*_loc_ as:

(21) $$I_{i}(F;S)=H_{i}(F)-H_{i}(F|S)$$

where, *I_i_* (F; *S*) denoted mutual information between the response and the spatial stimulus at the *i* ^th^ location (*i* = 1… *N*_loc_), and *F* defined the firing rate for *S*. The response entropy *H_i_* (F) was calculated as:

(22) $$H_{i}(F)=-\underset{j}{\sum^{\text{​}}}p_{i}\left(F_{j}\right)\log_{2}p_{i}\left(F_{j}\right)$$

where, *p_i_* (*F_j_* represented the probability of the firing rate lying in the *j* ^th^ response bin within the *i* ^th^ spatial location, and was computed as:

(23) $$p_{i}(F_{j})=\sum_{k=1}^{4}p_{i}(F_{j}|S_{k})p_{i}(S_{k})$$

In Eq. (23), *p_i_* (F_*j*_|S_*k*_) represented the conditional probability that the response was in the *j* ^th^ firing rate bin, given that the stimulus was in the *k* ^th^ spatial bin within the *i* ^th^ spatial location. *pi* (*Sk*) denoted the probability that the virtual animal was in the *k* ^th^ spatial bin within the *i* ^th^ spatial location, which was considered to follow a uniform distribution given the constant velocity assumption.

The noise entropy term *H_i_ (F*|*S*) in Eq. (21) was computed as:

(24) $$H_{i}(F|S)=\sum_{k=1}^{4}p_{i}\left(S_{k}\right)H_{i}\left(F|S_{k}\right)$$

where *H_i_* (F|s_k_) represented the conditional noise entropy for the *k* ^th^ spatial bin within the *i* ^th^ spatial location, calculated as:

(25) $$H_{i}\left(F|S_{k}\right)=-\underset{j}{\sum^{\text{​}}}p_{i}\left(F_{j}|S_{k}\right)\log_{2}\left(p_{i}\left(F_{j}|S_{k}\right)\right)$$

where *p_i_* (*F_j_|S_k_*) denoted the conditional probability of the firing rate being in the *j^th^* bin given that the stimulus was in the *K* ^th^ spatial bin within the *i* ^th^ location.

Together, this methodology of computing MI at several locations along the place field allowed us to assess spatial information transfer from all possible neural responses at that specific location. Note that *I_i_* (F; S), the mutual information computed for the *i* ^th^ spatial location is different from *I* (F; S), the *location-independent* mutual information that could be computed for the entire place field (spanning all firing rates and all spatial locations within the place field). We employed the location-dependent formulation *I_i_* (F; S) to compare this with stimulus-specific information metrics.

### 2.7 Spatial information transfer within a place field: Stimulus-specific information metrics

The second set of metrics that we used to compute spatial information transfer was derived from stimulus-specific information (SSI), obtained for 30 different trials of the entire traversal spanning all spatial locations. SSI has been proposed as a measure of information in neuronal response about a particular stimulus, and conveys the average specific information spanning all responses to a particular stimulus. To calculate the SSI, the spatial stimulus and the firing rate response were segregated into 80 and 40 bins, respectively. The SSI was calculated using the expression given below (Butts, 2003; Butts & Goldman, 2006; Montgomery & Wehr, 2010):

(26) $$SSI\left(S_{i}\right)=\sum_{j=1}^{40}p\left(F_{j}|S_{i}\right)I_{\text{sp}}\left(F_{j}\right)$$

where *p* (*F_j_|S_i_*) is the conditional probability of the firing rate being in the *j* ^th^ response bin given that the *i* ^th^ stimulus location was presented, and the *specific information I*_sp_ (*F*_j_) (DeWeese & Meister, 1999) was computed as:

(27) $$I_{\text{sp}}\left(F_{j}\right)=-\sum_{i=1}^{80}p\left(S_{i}\right)\log_{2}p\left(S_{i}\right)+\sum_{i=1}^{80}p\left(S_{i}|F_{j}\right)\log_{2}p\left(S_{i}|F_{j}\right)$$

Here, *p* (F_j_) is the probability of the firing rate being in the *j^th^* response bin and *p* (*S_i_|F_j_*) defined the conditional probability for the stimulus in the *i* ^th^ bin given that the firing rate was in the *j* ^th^ response bin. The first term in Eq. (27) represents the entropy of the stimulus ensemble *H*(*S*) and the second term represents the entropy of the stimulus distribution conditional on a particular firing rate response *H*(*S/F_j_*), providing *I*_sp_ (*F_j_* = *H (S) − H(S/F_j_*) (Butts, 2003; Butts & Goldman, 2006; Montgomery & Wehr, 2010). Thus, specific information defines the reduction in uncertainty about the spatial location gained by a particular firing rate response (*F*_j_), and *SSI* constitutes the average reduction of uncertainty gained from all firing rate responses given a particular spatial location (*S*_i_). As *I*_sp_ (*F*_j_ equals *I* (S; *F*_j_, the information gained from the observation of a specific output *F*_j_ about the range of possible spatial inputs *S*, the MI across the entire place field *I* (*S; F*) would be defined as $I(S;F)=\underset{j}{\sum^{\text{​}}}p\left(F_{j}\right)I\left(S;F_{j}\right)$ (DeWeese & Meister, 1999). Here, *p Fj* represents the probability of the firing rate lying in the *j* ^th^ response bin across the entire place field. As our focus in this study was on information metrics that were location-dependent (stimulus specific), we did not employ *I* (*S; F*), but have included the definition to illustrate the relationships and differences between *I*_sp_ (*F_j_*), *SSI* (S_i_), I_i_ (*F; S*), and *I* (*S; F*).

Bias in *I*_sp_ (*F_j_*) calculation was corrected using the Treves-Panzeri correction procedure (Bezzi et al., 2002; Montgomery & Wehr, 2010; Panzeri, Senatore, Montemurro, & Petersen, 2007; Panzeri & Treves, 1996; Treves & Panzeri, 1995) as follows:

(28) $$I_{\text{sp}-\text{corr}}\left(F_{j}\right)=I_{\text{sp}}\left(F_{j}\right)-C_{1}$$

where $C_{1}=\frac{\left(N_{\text{S}}-1\right)\left(N_{\text{R}}-1\right)}{2N_{\text{SRP}}\ln2}$ with *N*_S_ representing the total number of stimulus bins, *N*_R_ denoting the total number of response bins and *N*_SRP_ depicting the total number of stimulus–response pairs.

Spatial information transfer as a function of space within a place field was found to be bimodal or trimodal in several scenarios. To quantify the information and compare the information transfer across models and across the different levels of trial-to-trial variability, several MI-based and SSI-based information metrics were developed (listed in Table 3).

### 2.8 Exploring parametric dependencies in spatial information transfer

A single hand-tuned model does not account for the numerous biophysical heterogeneities inherent to neural structures, and the results obtained with a single model could be biased by the specific selection of parametric values. A simple methodology to account for the biophysical heterogeneities with signature electrophysiological properties of specific neuronal subtype under consideration is to build a population of models. We employed a multi-parametric multi-objective stochastic search (MPMOSS) algorithm to arrive at a population of models that would satisfy the several biophysical heterogeneities (by allowing the multiple parameters to span an experimental range, shown in Table 1) and would match with bounds on several electrophysiological measurements (Table 2). Since this procedure involves a uniform random sampling of parameter values, it is unbiased and provides a good strategy to search for interdependencies between parametric combinations that yield signature electrophysiological characteristics.

To match physiological outcomes, these models were then validated on the basis of sharpness of their place-cell firing properties (*F*_max_ > 56 Hz and *FWHM* < 2.5 s; 2 measurements), six signature intraneuronal functional maps (Basak & Narayanan, 2018, 2020; Narayanan & Johnston, 2012) of back propagating action potential amplitude (*bAP*), input resistance (*R*_in_), resonance frequency (*f*_R_), maximum impedance amplitude (|*Z*|_max_), strength of resonance (*Q*) and total inductive phase (Φ_L_), each validated at three locations (soma, ~ 150 μm and ~ 300 μm from soma on the apical trunk; total 18 measurements) and firing rate at the soma resulting from step current injections of 100 pA, 150 pA, 200 pA and 250 pA (4 measurements). Only the models that matched the bounds on these 24 measurements (Table 2) were declared valid. To explore interdependencies among parameters that resulted in the valid models, which showed sharp place-field tuning and manifested signature intrinsic electrophysiological properties, pairwise Pearson’s correlation coefficients spanning the parameters of all valid models were computed. To assess the impact of individual channels in the model on spatial information transfer, we removed each channel individually from the model (by setting the conductance value associated with that channel to zero) and assessed how the information measures changed due to the removal of this ion channel.

### 2.9 Computational details

All simulations were performed using custom-written software in the NEURON simulation environment (Carnevale & Hines, 2006), at 34 °C with an integration time step of 25 μs. Unless otherwise stated, all simulations were performed with a resting potential of –65 mV. Analysis was performed using custom-built software written in Igor Pro programming environment (Wavemetrics). Statistical tests were performed using statistical computing language R (www.R-project.org), and the *p* values are reported while presenting the results, or in the respective figure panels or associated captions. In qualitatively defining weak and strong correlations, we followed the nomenclature introduced by (Evans, 1996) by placing thresholds on the absolute value of the Pearson’s correlation coefficient: 0–0.19: Very weak; 0.2–0.39: Weak; 0.4–0.59: Moderate; 0.6–0.79: Strong; 0.8–1: Very Strong. To avoid potential misinterpretations arising from representing data by merely their summary statistics (Marder & Taylor, 2011; Rathour & Narayanan, 2019), all data points from the population of neural models are depicted as beeswarm or scatter plots.

## 3 Results

We built a morphologically realistic, conductance-based model of a CA1 pyramidal cell, incorporating electrophysiologically characterized passive and active mechanisms (Fig. 1A). The model contained 10 distinct biophysically constrained ion channel subtypes that were distributed along the somatodendritic arbor to match experimental findings (Fig. 1B). We hand-tuned the base model parameters (Table 1) to match several intrinsic somatodendritic electrophysiological properties (Table 2) of rat CA1 pyramidal neurons (Fig. 1C–H). We tuned the strength of synaptic connections such that the somatic unitary AMPAR EPSP was set to ~0.2 mV (Fig. 1I) irrespective of synaptic location within the *stratum radiatum* of the CA1 pyramidal neuron (~350 μm of apical dendrites from the soma).

### 3.1 Ion-channel degeneracy in the concomitant emergence of sharply tuned spatial firing profile and intrinsic physiological properties of the neuron

As a first step in evaluating the impact of heterogeneous ion channel combinations on sharp tuning of place-cell responses, we generated 12,000 random models by independent selection of parameter values from their respective uniform distributions (Table 1). We randomly dispersed 80 distinct synaptic locations (of the 428 possible locations) across the *stratum radiatum* where presynaptic afferent inputs impinged. These 80 synapses received independent presynaptic inputs governed by Eq. (17), and the somatic voltage response of the neuron was recorded to compute the place-field firing rate profile.

We validated the firing rate profiles of these randomly generated neuronal models for sharpness of place field tuning by placing thresholds on maximum firing rate within the place field (> 56 Hz) and the width of the firing rate profile (<2.5 s), and found 1024 of the 12,000 models (~8.5%) to satisfy these constraints (Fig. S1). We picked five models within these 1024, with similar place-field firing profiles reflected as similar values of *F*_max_ and *FWHM* and asked if similar place-field tuning required similar parametric combinations (Fig. S1A–B). Consistent with prior findings with models endowed with fewer ion channels (Basak & Narayanan, 2018, 2020), we found evidence for ionchannel degeneracy in the expression of sharp place-field tuning (Fig. S1C). Across all 1024 sharply-tuned models, whose *F*_max_ and *FWHM* are depicted in Fig. S1D–E, the parameters spanned the entire valid range of parameters pointing to the absence of any parametric clustering in arriving at sharp spatial tuning (Fig. S1F). We explored pairwise correlations of the parameters underlying these place-cell models with sharply tuned firing profiles, and found most of the correlation coefficients to be weak (Fig. S1F).

**Fig. 2:**
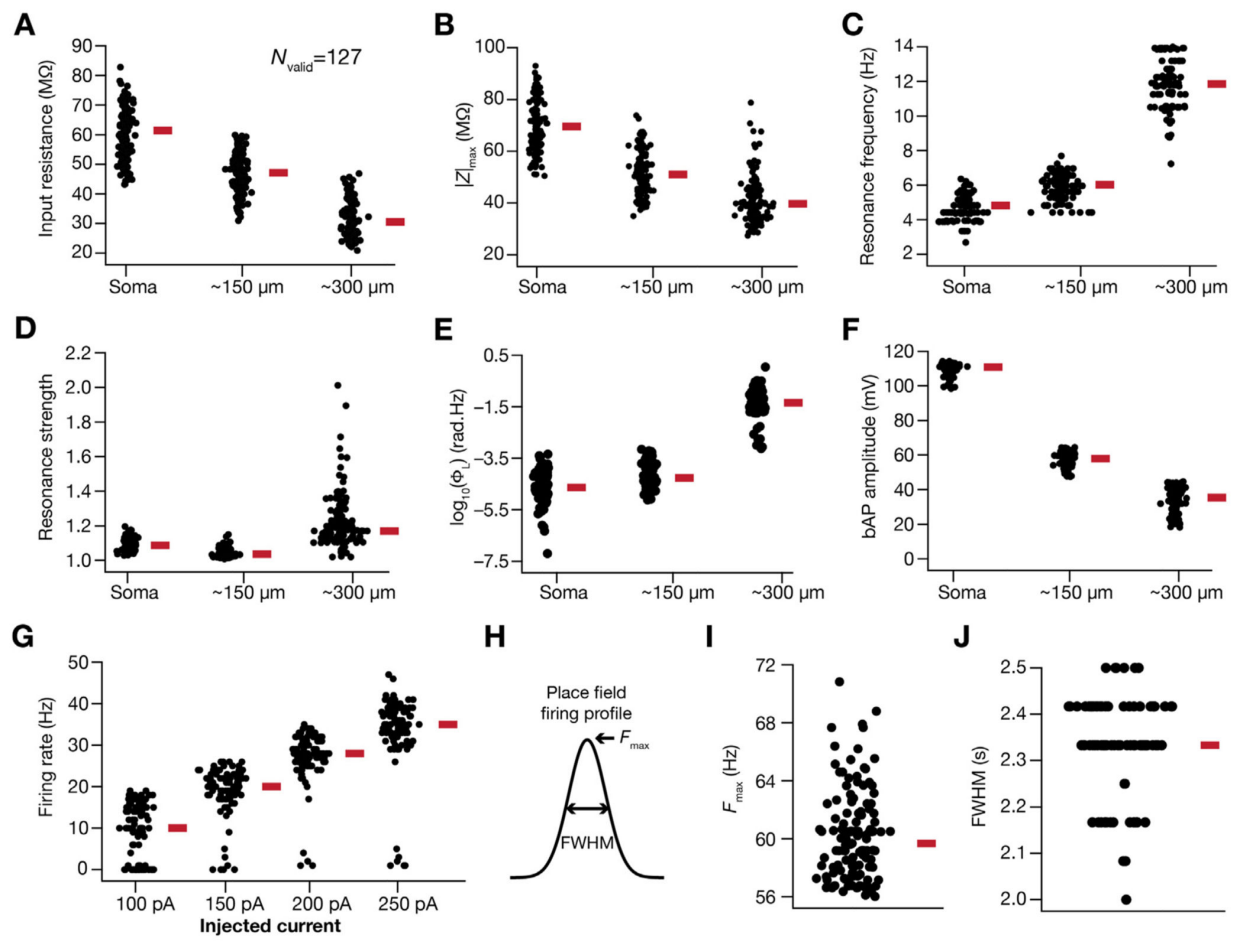
A subset of models generated through a stochastic search process showed sharp place-cell tuning and manifested signature somatodendritic intrinsic measurements of CA1 pyramidal neurons. Out of 12000 randomly generated models, 127 satisfied 20 intrinsic somatodendritic measurements and manifested sharply-tuned place field firing. (A–G) The intrinsic measurements for the 127 valid models are shown: input resistance (*R*_in_, A), maximum impedance amplitude (*|Z|*_max_, B), resonating frequency (*f*_R_, C), strength of resonance (*Q*, D), total inductive phase (Φ_L_, E) and backpropagating action potential (bAP) amplitude (F), each of them at three locations (soma, ~150 μm from soma and ~300 μm from soma) on the apical trunk; and the firing rate for step currents of 100 pA, 150 pA, 200 pA and 250 pA at the soma (G). (H) A typical place-field firing profile illustrating the measurement of maximum firing rate (*F*_max_) and the temporal distance between the places with half the maximum value of firing rate (*FWHM*). A relative criterion on tuning sharpness, involving high *F*_max_ (>56 Hz) and low *FWHM* (<2.5 s), was applied to obtain the 127 valid place-cell models (out of the 12000 randomly generated models). (I–J) Place field firing measurements *F*_max_ and *FWHM* at the soma for the 127 models.

**Fig. 3:**
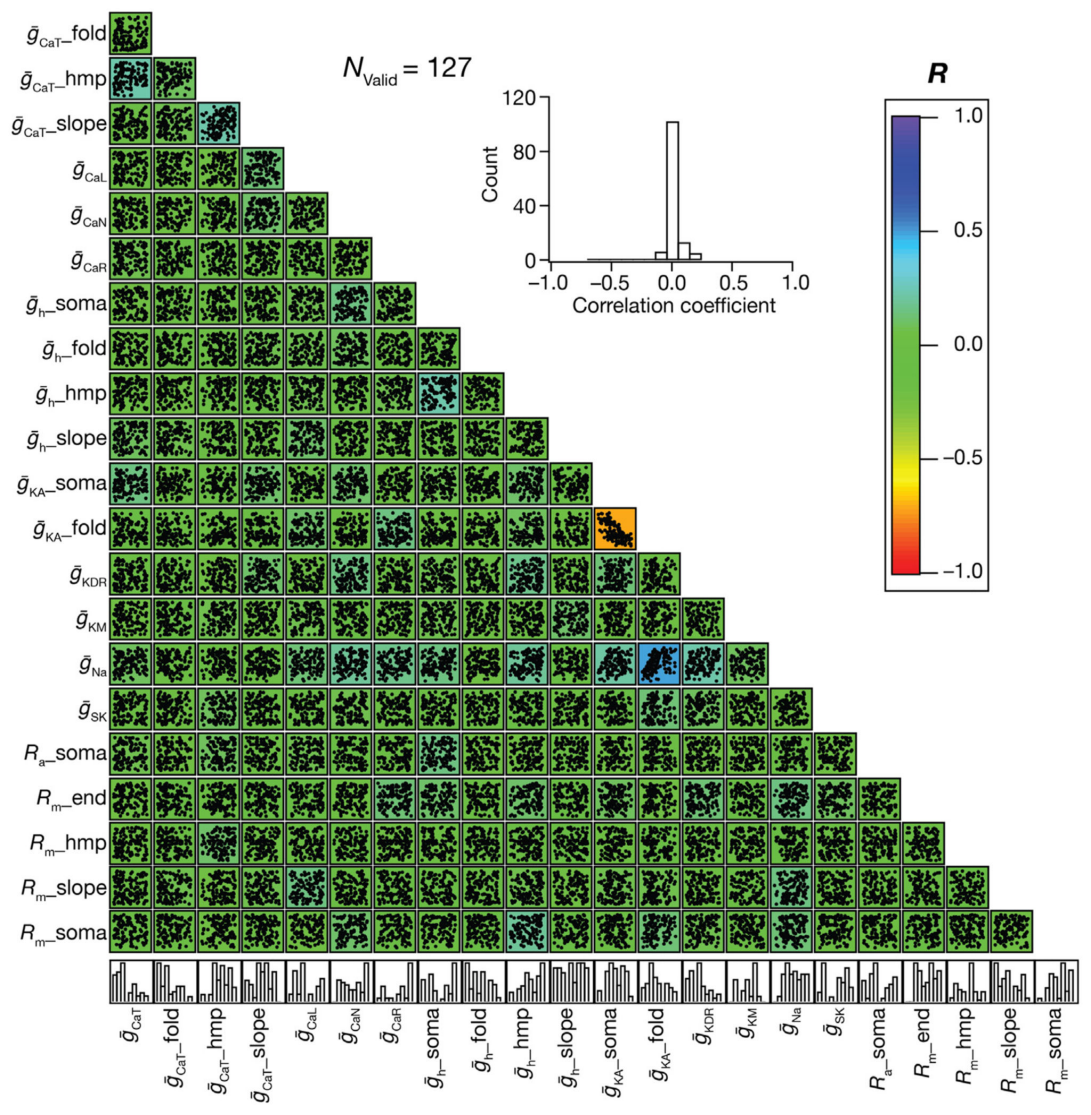
Models showing sharp place-field firing and signature intrinsic characteristics exhibited wide parametric variability and weak pair-wise correlations among underlying parameters. Pairwise scatter plot matrix of parametric values defining the 127 valid models superimposed on the corresponding correlation coefficient matrix. Inset shows the histogram of all the correlation coefficient values.

Whereas place-field tuning constitutes one aspect of CA1 pyramidal neuron physiology, their well-characterized signature somatodendritic intrinsic properties form defining electrophysiological attributes. To match our model population with these signatures, we validated the 1024 sharply tuned models against 22 distinct electrophysiological measurements (Table 2): each of input resistance, backpropagating action potential amplitude, maximal impedance amplitude, resonance frequency, resonance strength and total inductive phase at 3 different somatodendritic locations; and action potential firing rate in response to somatic pulse current injections at 4 different current values. Of the total 12,000 models generated, we found 127 (~1.06%) models to match all 24 measurement bounds (Table 2) and were declared valid. We picked five models within these 127 valid models, with similar place-field firing profiles (Fig. S2 A) and similar intrinsic measurements across the somatodendritic axis (Fig. S2B–F). We assessed the parameters associated with five models and found evidence for ion-channel degeneracy in the concomitant expression of sharp place-field tuning and signature intrinsic properties (Fig. S2G). Across all 127 models that were intrinsically-valid (Fig. 2A–G) and sharply-tuned (Fig. 2H–J), the parameters spanned the entire valid range of parameters pointing to the absence of any parametric clustering in these models (Fig. 3). We explored pairwise correlations of the parameters underlying these models, and found most of the correlation coefficients to be weak (Fig. 3).

Together, the unbiased stochastic search procedure provided us with a population of place-cell models that exhibited several signature electrophysiological properties, and manifested sharp place-field tuning in their firing rate profiles. We employed this population of place-cell models for assessing the impact of several biophysical and physiological characteristics on spatial information transfer within the place field.

### 3.2 Heterogeneities in the regulation of spatial information transfer by trial-to-trial variability in place-cell responses

**Fig. 4:**
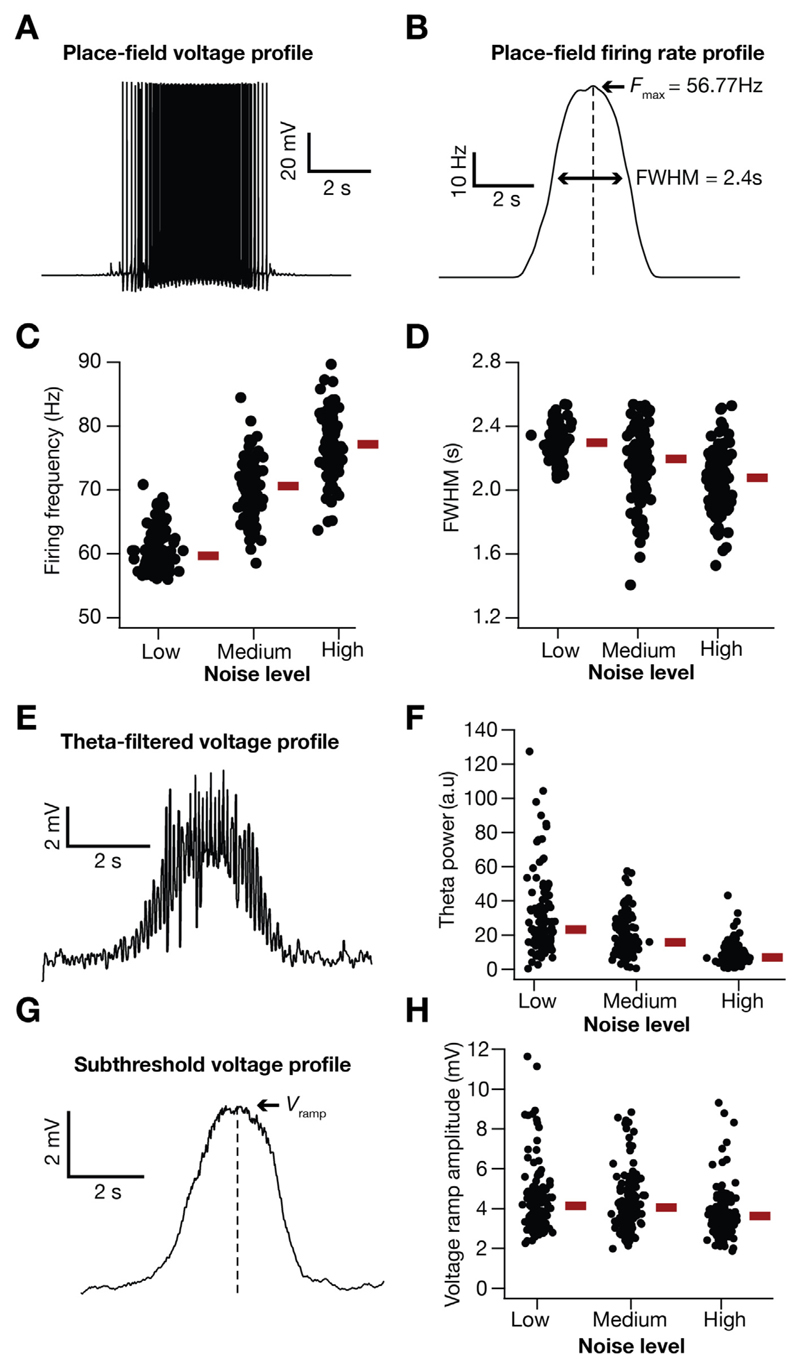
Impact of additive Gaussian white noise (AGWN) on place-cell characteristics. (A–B) Voltage trace (A) and corresponding firing rate profile (B) during traversal of a place field in a typical valid place-cell model in the presence of AGWN (σ_noise_ = 5×10^−4^ Hz^2^). (C–D) Impact of different levels of AGWN on the peak firing frequency, *F*_max_ (C) and full-width at half maximum, *FWHM* (D) of the 127 valid place-cell models. The red bars represent the respective median values. *F*_max_: Kruskal Wallis test, *p =* 2.2×10^−12^, Wilcoxon Signed Rank test, Low *vs*. Medium *p =* 3.6×10^−8^, Medium *vs.* High *p =* 5.3×10^−6^, Low *vs.* High = 3.3×10^−10^. FWHM: Kruskal Wallis test, *p =* 8.8× 10^−8^, Wilcoxon Signed Rank test, Low *vs*. Medium *p =* 4.3×10^−4^, Medium *vs.* High *p =* 2.3×10^−4^, Low *vs.* High = 5.3×10^−6^. (E) Voltage profile in (A) filtered to emphasize theta-frequency oscillations during traversal of a place field. (F) Impact of different levels of AGWN on theta power of the 127 valid place-cell models. Kruskal Wallis test, *p* = 1.2× 10^−8^, Wilcoxon Signed Rank test, Low *vs*. Medium *p* = 7.0×10^−4^, Medium *vs.* High *p* = 6.1× 10^−7^, Low *vs.* High = 5.3× 10^−8^. (G) Voltage profile in (A) filtered to emphasize subthreshold voltage ramp during traversal of a place field. (H) Impact of different levels of AGWN on voltage ramp amplitude of the 127 valid place-cell models. Kruskal Wallis test, *p*=2×10^−4^, Wilcoxon Signed Rank test, Low *vs*. Medium *p =* 0.4152, Medium *vs.* High *p =* 3.4×10^−3^, Low *vs.* High = 7.7×10^−5^. When present, the red bars represent the respective median values. AGWN σ_noise_ values: *Low*: 5×10^−4^ Hz^2^, *Medium:* 1×10^−3^ Hz^2^, *High:* 5×10^−3^ Hz^2^.. (For interpretation of the references to color in this figure legend, the reader is referred to the web version of this article.)

The firing profile of a place cell within its place field represents a spatial tuning curve. For instance, in a symmetric firing profile (*e.g.*, Fig. 4A–B), the spatial location at the center of the place-field elicits the peak firing response and the response progressively reduces for spatial stimuli on either side of this peak. Within the place field of this neuron, does maximal spatial information transfer occur at the peak of this tuning curve or at the high-slope regions of the tuning curve? Prior studies in other brain regions have shown that the answer to this question depends on several factors, with trial-to-trial variability playing a prominent role in regulating the relationship between the tuning curve and information transfer (Butts & Goldman, 2006; Montgomery & Wehr, 2010). To address this question for spatial information within the place field of individual place cells, we incorporated trial-to-trial variability in neural responses by introducing noise into the afferent input rate (Eq. (19)).

**Fig. 5:**
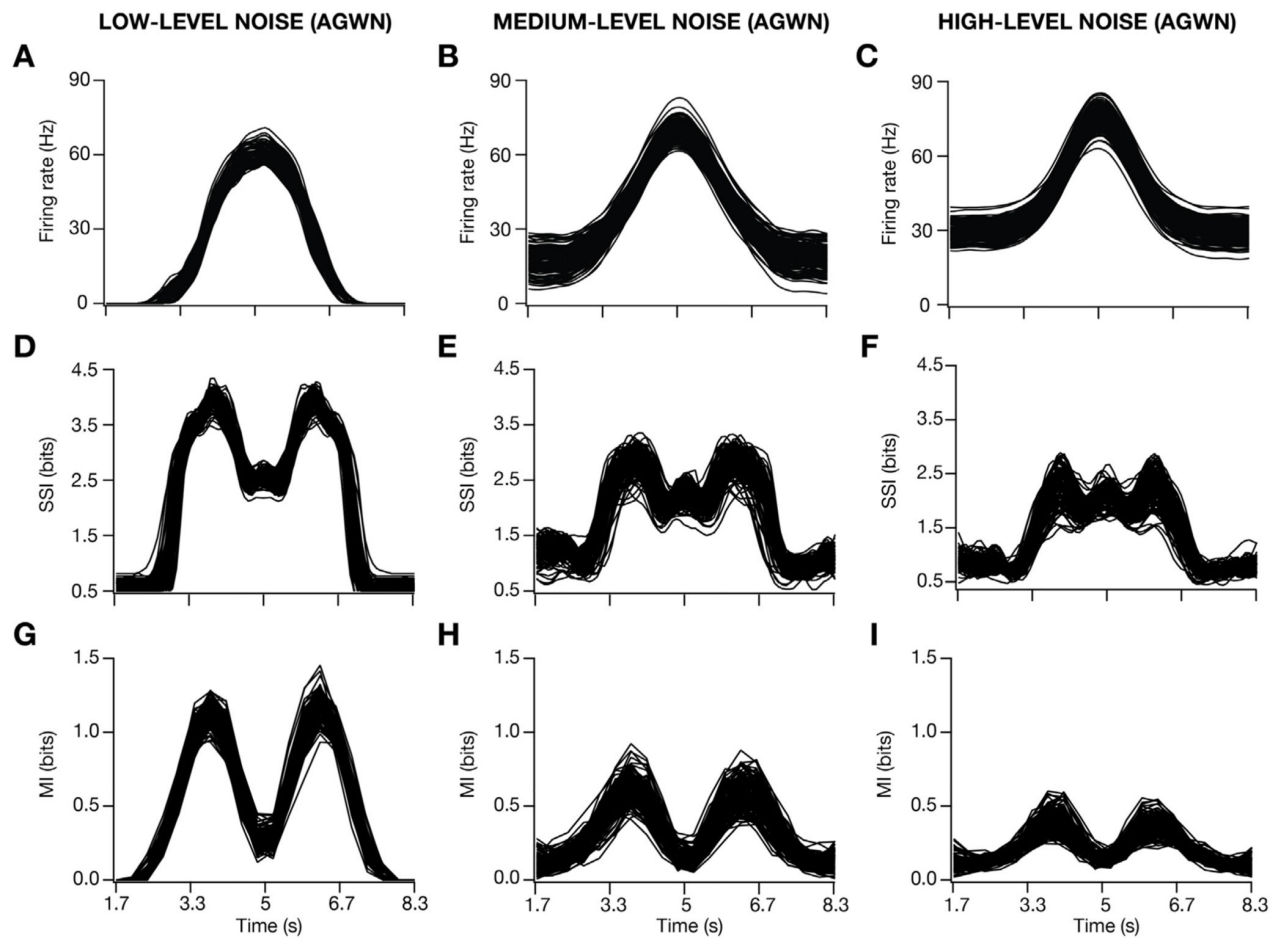
Enhanced trial-to-trial variability, imposed as an additive Gaussian white noise (AGWN), reduced spatial information transfer in place-cell models. (A–I) Firing rate profiles (A–C), stimulus specific information (SSI) profiles (D–F), and mutual information profiles (G–I) as functions of time, shown for low (plots on the left), medium (plots in the middle), high (plots on the right) levels of AGWN. AGWN σ_noise_ values: Low: 5×10^−4^ Hz^2^, Medium: 1×10^−3^ Hz^2^, High: 5×10^−3^ Hz^2^.

The introduction of input noise as additive Gaussian white noise (AGWN) manifested as trial-to-trial variability in the firing rate responses, enhanced the firing rate (Fig. 4C) and reduced the width (Fig. 4D) of place-cell responses. Across all 127 valid models, progressive increase in trial-to-trial variability, introduced by increasing σ_noise_ (Eq. (19)), resulted in a progressive increase in the peak firing rate (Fig. 4C), and progressive reductions in the FWHM (Fig. 4D), theta power (Fig. 4E–F) and the voltage ramp (Fig. 4G–H) of the place-field response profile. We performed 30 trial simulations for each of the 127 valid place-cell models, obtained their firing rate profiles for 3 different levels of noise (Fig. 5A–C; designated as low, medium and high) and computed stimulus-specific information (SSI; Fig. 5D–F) and mutual information (MI; Fig. 5G–I) for all these 127 models.

We noted marked heterogeneity in spatial information, assessed with the SSI and MI profiles across models (Fig. 5D–I). Importantly, at low levels of trial-to-trial variability, the SSI (Fig. 5D) and the MI (Fig. 5G) showed maximal spatial information transfer at the high-slope locations of the corresponding spatial tuning curves (Fig. 5A). Consequently, both the SSI and the MI profiles were bimodal when low levels of trial-to-trial variability was introduced, although the values of SSI at high-firing locations were higher compared to MI values at these locations. With increased trial-to-trial variability, introduced as AGWN, the out-of-field firing rates increased (Fig. 5B–C) while also enhancing the peak firing rate (Fig. 5B–C; Fig. 4C).

Progressively enhancing trial-to-trial variability by increasing σ_noise_ resulted in a marked reduction in spatial information across models, while still manifesting heterogeneity in spatial information transfer across the model population (Fig. 5E–F; Fig. 5H–I). Whereas the MI profile maintained bimodality despite reduction in the transferred information with higher levels of trial-to-trial variability (Fig. 5H–I), there was a progressive transition from a bimodal (Fig. 5D) to a trimodal (Fig. 5E–F) distribution of the SSI profiles. The transition in the SSI profile was consequent to the suppression in spatial information transfer at the high-slope locations of the tuning curve, with relatively small changes to spatial information transfer at the high-firing locations (Fig. 5D–F).

**Fig. 6:**
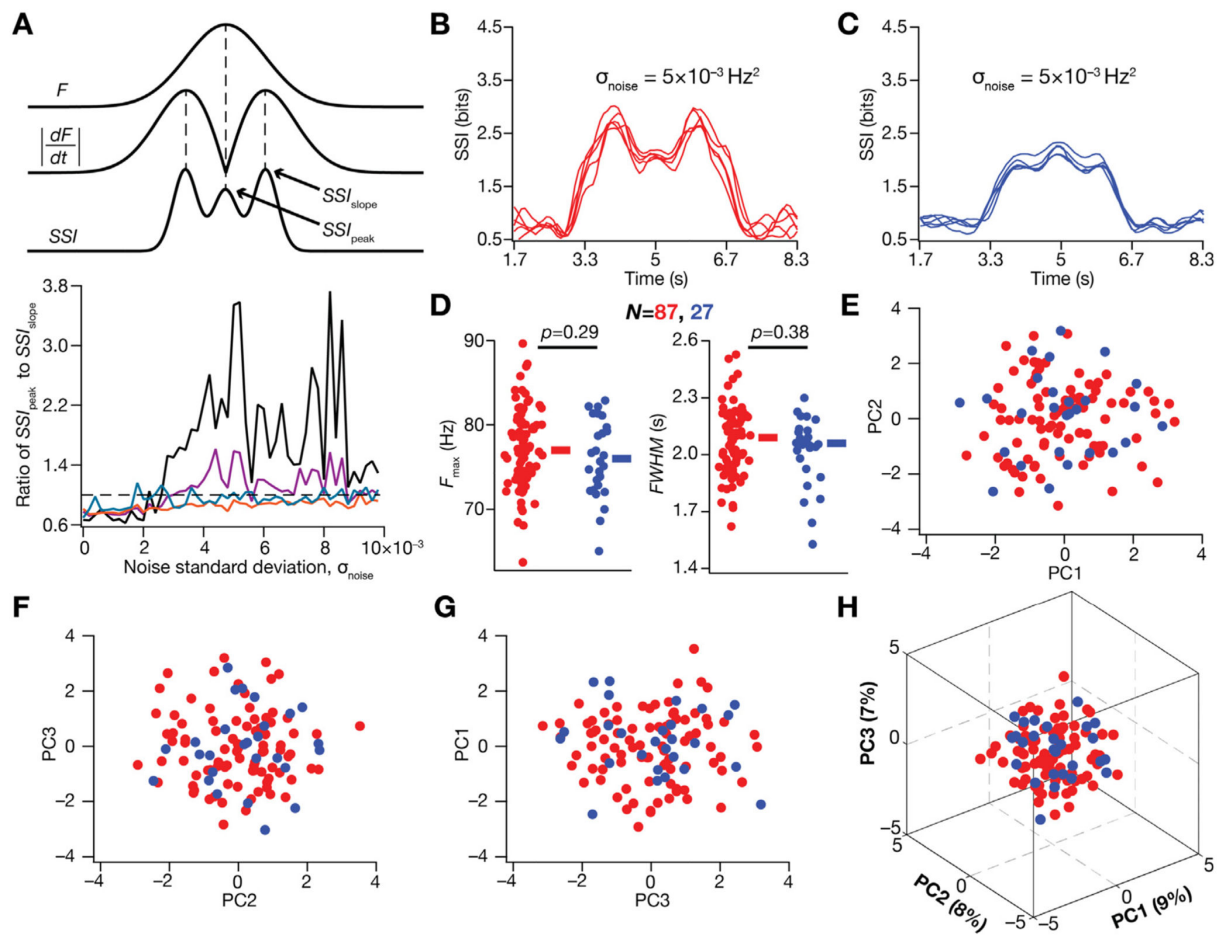
Heterogeneous impact of enhanced trial-to-trial variability on spatial information transfer in place cells. (A) Top, Illustration of the measurements *SSI*_peak_ and *SSI*_slope_. *SSI*_peak_ depicts the SSI value at the location where the place-field firing profile (F) is at its peak, and *SSI*_slope_ represents the SSI value at the location where the absolute slope of the place-field firing profile, $\left|\frac{dF}{dt}\right|$, is at its peak. *Bottom*, Traces from four representative models showing the heterogeneity in the evolution of *SSI*_peak_/*SSI*_slope_ as a function of enhanced trial-to-trial variability. (B–C) There were broadly two classes of models, one where the *SSI*_peak_ was low even at high noise levels (B; several representative examples shown in red), and another where *SSI*_peak_ was the highest SSI when noise level was high (C; several representative examples shown in blue). (D) Peak firing rate (left) and FWHM (right) of the two classes of model subpopulations. The rectangles besides each plot represent the respective median value. σ_noise_ = 5×10^−3^ Hz^2^. p values provided correspond to the Wilcox rank sum test. (E–H) Principal component analyses on the parameters underlying the two classes of models shown in B (red) and C (blue). Shown are the coefficients associated with these model parameters with reference to the first three principal components. The percentage variance explained by each principal component is provided within parentheses in panel H.. (For interpretation of the references to color in this figure legend, the reader is referred to the web version of this article.)

To further assess this transition in the SSI profile with enhanced trial-to-trial variability, we increased σ_noise_ to larger values and computed the values of the SSI at the high-slope locations (*SSI*_slope_, the average value from the two peaks of the SSI, computed for symmetric firing profile; Fig. 6A) and at the peak-firing locations (*SSI*_peak_; Fig. 6A). We computed the ratio *SSI*_peak_/*SSI*_slope_ and plotted this as a function of σ_noise_ (Fig. 6A). A value less than unity for this ratio indicates that maximal stimulus specific spatial information was transferred at the high-slope regions, whereas a value above unity reflects maximal SSI at the peakfiring location. Whereas *SSI*_peak_/*SSI*_slope_ was less than unity for low values of σ_noise_ across all models (Fig. 5D, Fig. 6A), two sub-populations of models emerged with higher values of σ_noise_. In one subpopulation (*N* = 87), *SSI*_peak_/*SSI*_slope_ was always lower than unity even with higher levels of trial-to-trial variability (teal and orange plots in Fig. 6A, bottom panel; example SSI profiles in Fig. 6B); in a second smaller subpopulation (*N* = 27), this ratio was less than unity for low levels of trial-to-trial variability but transitioned to values higher than unity for higher levels of trial-to-trial variability (black and purple plots in Fig. 6A, bottom panel; example SSI profiles in Fig. 6C). Thus, whereas a large proportion of models transferred maximal spatial information at the high-slope locations irrespective of the level of trial-to-trial variability, a subpopulation of models switch to transferring maximal information at the peak-firing locations with higher levels of trial-to-trial variability.

We found that there were no significant differences in the peak firing rate or the width of the place-field firing profiles of models within the two model subpopulations, the ones showing higher SSI at high-slope *vs*. high-firing locations with high levels of trial-to-trail variability (Fig. 6D). Were there systematic differences in the parameters that defined models within these two subpopulations? To answer this question, we performed principal component analysis (PCA) on parameters that governed the models within the two subpopulations (Fig. 6E–H). We asked if there were distinct clusters representative of the two subpopulations in the reduced dimensional space, pointing to structured parametric differences between these two populations. We found that the three principal dimensions explained merely 24% of the total variance, and there was considerable overlap in the coefficients associated with these two subpopulations, suggesting the absence of systematic parametric differences in the subpopulations (Fig. 6E–H).

**Fig. 7:**
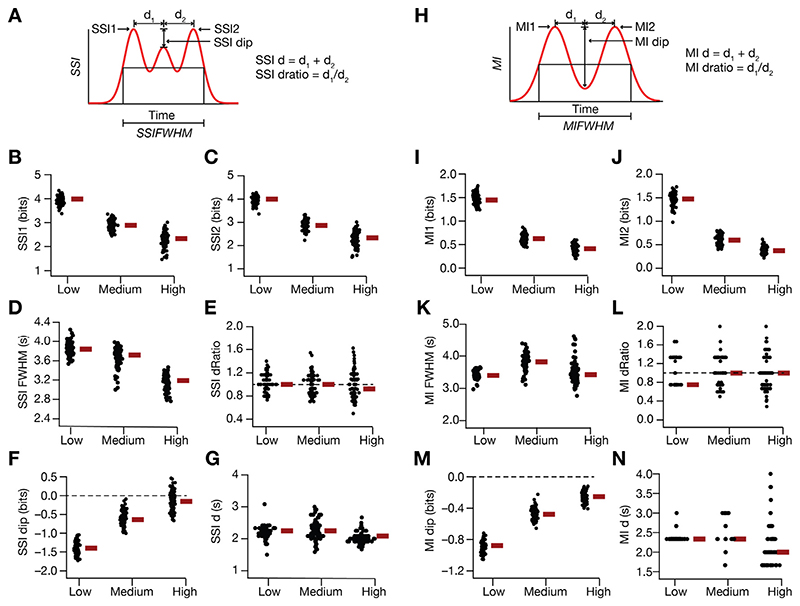
Quantification of the reduction in spatial information transfer as a consequence of enhanced trial-to-trial variability, imposed as an additive Gaussian white noise (AGWN) in place-cell models. (A) Idealized representation of stimulus-specific information (SSI) as a function of time, illustrating the various metrics developed here for quantifying spatial information transfer in place cell models. (B–G) SSI metrics for the population of valid models depicting the impact of three levels of noise on the first (B, SSI1) and second (C, SSI2) peaks of SSI, the full width half maximum of the SSI profile (D, SSIFWHM), the ratio of the first peak-to-center distance to the center-to-second peak distance (E, SSI dRatio), the difference between the SSI value at the place field center to the peak SSI value (F, SSI dip) and the difference between the location of SSI1 and SSI2 (G, SSI d). (H–N) Same as (A–G) for mutual information profiles of the valid model population. AGWN σ_noise_ values: *Low:* 5×10^−4^ Hz^2^, *Medium:* 1×10^−3^ Hz^2^, *High:* 5×10^−3^ Hz^2^.

We developed 12 distinct profile-specific metrics for quantifying the SSI (Fig. 7A) and MI (Fig. 7H) profiles for the 127 models for three levels of noise. These quantitative metrics confirmed the considerable heterogeneities in spatial information transfer across the model population (Fig. 7). These results showed that across models, information transferred reduced with increase in trial-to-trial variability, with symmetry in spatial information transfer at the two-high slope regions (Fig. 7B–C, Fig. 7I–J). These quantitative metrics also corroborated the emergence of the two subpopulations (Fig. 6) at high values of σ_noise_; specifically, the value of *SSIdip* (Fig. 7F) was greater than zero in a small sub-population of models, indicating that these models transfer maximal information at the peak-firing location compared to the high-slope locations (Fig. 7A). The value of *MIdip* (Fig. 7M), however, was always negative across all measured values of σ_noise_.

### 3.3 Spatial information transfer in neurons with multiple presynaptic place-field inputs onto the CA1 pyramidal neuron with white or pink noise

**Fig. 8:**
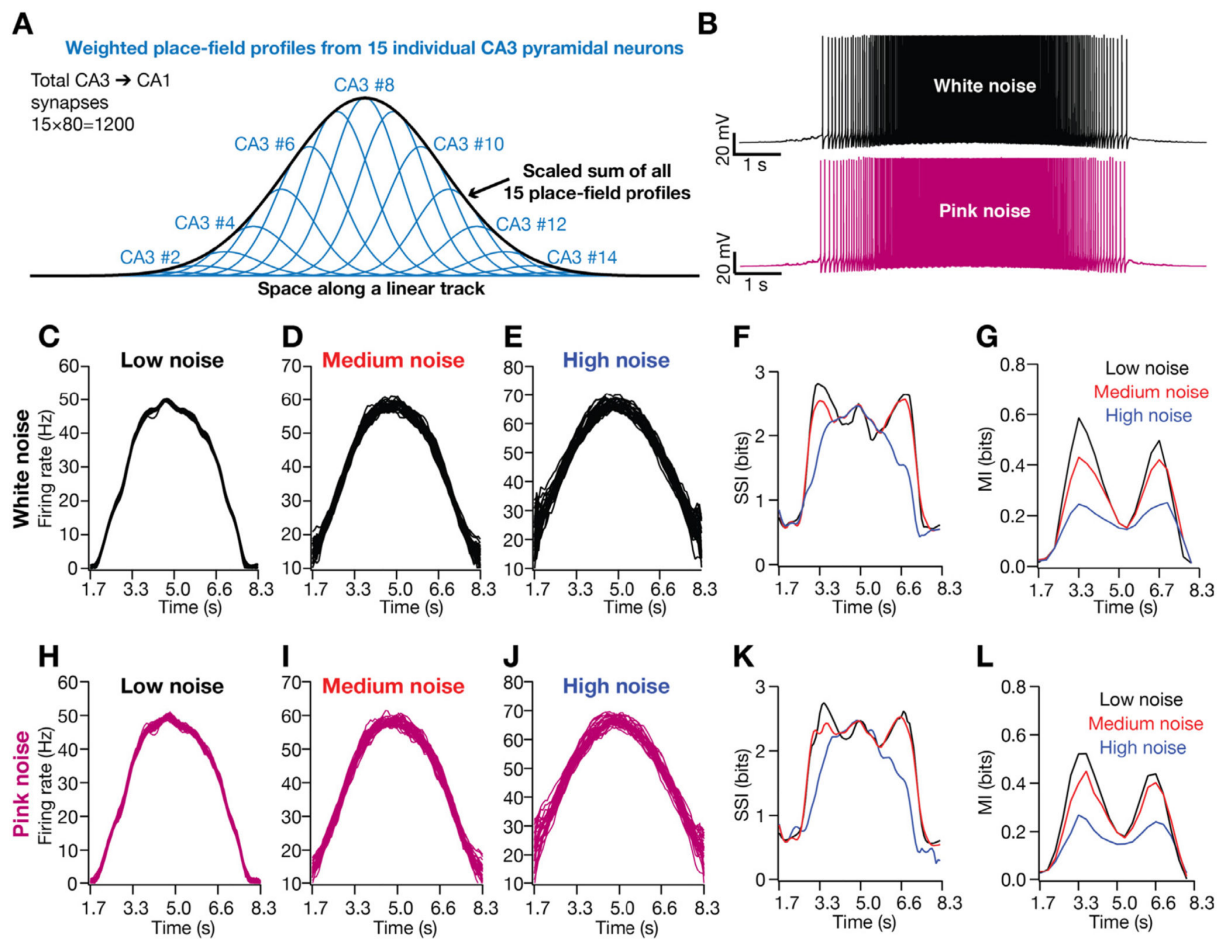
Firing rate and profiles of spatial information transfer in neurons receiving inputs from multiple CA3 pyramidal neurons, in the presence of white or pink additive noise. (A) Schematic representation of the place-field profiles of 15 presynaptic CA3 pyramidal neurons weighted by their synaptic strength (blue traces). Each of these presynaptic neurons made 80 synapses on the postsynaptic CA1 pyramidal neuron, yielding a total of 80×15 = 1200 synapses from the presynaptic ensemble to the postsynaptic neuron. These synapses were postsynaptically randomly dispersed across the *stratum radiatum* of the morphologically realistic CA1 pyramidal neuron. The sum of all the 15 weighted presynaptic profiles is shown (thick black trace) follows a Gaussian profile enabling the formation of a symmetric place field of the postsynaptic neuron. (B) Example voltage response of the CA1 pyramidal neuron receiving inputs from the 15 CA3 pyramidal neurons shown in panel A, in the presence of Gaussian white noise (black) or pink noise (pink). (C–E) Firing rate profiles of the CA1 pyramidal neuron receiving inputs from the 15 CA3 pyramidal neurons shown in panel A, in the presence of low (C), medium (D) and high (E) levels of AGWN. Each panel shows the firing rate profile for all the 30 trials of simulations. (F–G) Spatial information transfer profiles, computed either as SSI (F) or MI (G), from the firing rate profiles shown in panels C–E for the three levels of AGWN (Black: low noise, Red: medium noise, Blue: High noise). (H–L) Same as panels (C–G), but in the presence of additive pink noise. σ_Noise_ values: *Low*: 5×10^−4^ Hz^2^, *Medium*: 1×10^−3^ Hz^2^, *High*: 5×10^−3^ Hz^2^.. (For interpretation of the references to color in this figure legend, the reader is referred to the web version of this article.)

The formulations in Eqs. (17)–(18) for presynaptic spike train generation within a single place field of the postsynaptic neuron implemented probabilistic activation of the presynaptic neurons within a single postsynaptic place field. These formulations did not account for the different presynaptic neurons, each endowed with heterogeneous place field locations and differential synaptic weights in connecting to the postsynaptic neuron (Bittner et al., 2015, 2017; Grienberger et al., 2017). However, the summation of the probabilities of firing of each presynaptic neuron, weighted by their respective synaptic strengths (which mimics a Gaussian centered at the place-field center of the postsynaptic neuron) would result in a probability distribution that is approximated by a Gaussian with appropriate scaling factor and standard deviation (Seenivasan & Narayanan, 2020; Fig. 8A). Thus, the probabilistic formulation of presynaptic firing should be interpreted as that of a population of presynaptic neurons, each with differential synaptic strengths and heterogeneous place-field locations, converging on the postsynaptic structure (Seenivasan & Narayanan, 2020).

The equivalence of our probabilistic formulation of synaptic inputs within a single place field to heterogeneous presynaptic inputs from multiple CA3 pyramidal neurons (with appropriate synaptic weights) is exact in a single-compartmental model (Seenivasan & Narayanan, 2020). However, in a multicompartmental model, owing to spatial distribution of synapses and the presence of dendritic nonlinearities, the equivalence could be hampered. To address this, we simulated spatially modulated spike trains from 15 different CA3 pyramidal neurons with heterogeneous place fields to impinge on the postsynaptic neuron (Fig. 8A). Each of these 15 presynaptic neurons made 80 randomly dispersed synaptic contacts (AMPAR-NMDAR synapses) on the *stratum radiatum* of the CA1 pyramidal neuron, making a total of 80 × 15 = 1200 synapses.

Consistent with prior experimental analyses and computational studies (Bittner et al., 2015, 2017; Grienberger et al., 2017), we assigned the strength of the synapses from individual CA3 neurons to follow a Gaussian profile based on their place-field position along the virtual arena. This was implemented by scaling the permeability values (*P̄_AMPAR_* and *P̄_NMDAR_*) of the synapses according to the spatial location of the corresponding presynaptic CA3 neuron (Fig. 8A). We computed the neural responses in the presence of such synaptic activation (Fig. 8B) in the presence of low, medium or high level of AGWN (Fig. 8C–E), and found SSI (Fig. 8F) and MI (Fig. 8G) profiles to be quantitatively and qualitatively similar to those obtained with synaptic activation profiles in Eqs. (17)–(18).

Although we have incorporated Gaussian white noise in our simulations to model trial-to-trial variability, biological noise typically manifests 1/*f* characteristics (pink noise) in the frequency domain (Buzsaki, 2006; Gilden, 2001; Gisiger, 2001; Hausdorff & Peng, 1996; Ward, 2001). To account for this, we modeled trial-to-trial variability as pink noise, generated as a low-pass filtered version of the Gaussian white noise. Although there were minor differences in terms of the exact values of firing rate profiles (Fig. 8H–J) and the information transfer profiles (Fig. 8K–L), broadly our conclusions about SSI and MI profiles were similar with white or pink noise profiles (Fig. 8).

### 3.4 Degeneracy in the emergence of place cells manifesting similar rate-based spatial information transfer profiles

We computed the SSI and MI profiles for the five similar models shown in Fig. S2, and found they possessed similar SSI and MI metrics as well (Table S1). The parametric values of these similar models, however, were distributed over the entire span of the respective parametric space (Fig. S2G). These point to the expression of degeneracy in concomitantly achieving similar intrinsic properties and similar rate-based spatial information transfer in place cells.

In further exploring the dependencies of spatial information transfer on model parameters, we asked if any of the model parameters values would predict spatial information transfer with different levels of trial-to-trial variability. To answer this, we computed pairwise correlations between 20 physiological measurements (3 somatodendritic measurements of *R*_in_, | *Z*|_max_, *f*_R_, *Q*, *Φ_L_* and bAP; *F*_max_ and FWHM for place-field profiles in the absence of noise) that defined the 127 valid models and the 12 information transfer measurements (Table 3) that were obtained from the place-field responses of these models with low (Fig. S3), medium (Fig. S4) and high (Fig. S5) levels of trial-to-trial variability. Although there were expected strong correlations between some of the information metrics – such as strong positive correlations between *SSI1 vs. SSI2* and *SSI1/SS2 vs. MI1/MI2*, and strong negative correlations between *SSI1/SSI2 vs. SSIdip* across all three values of σ_noise_ – the pairwise correlations between information metrics and model measurement values were weak (Fig. S3–S5).

Our outcomes thus far froze synaptic locations at one specific randomized localization and varied ion channel conductances exploring parametric dependencies of spatial information transfer. In another set of simulations, we varied localization of the 80 distinct synapses along the dendritic arbor in the base model (Table 1; Fig. 1). Specifically, we randomly dispersed the 80 synapses across the apical dendritic arbor to 400 combinations of distinct locations, computed the firing rate profile and the information transfer profiles and plotted the associated measurements (Fig. S6). We found that the introduction of heterogeneities in synaptic localization profiles introduced heterogeneities in spatial firing profiles (Fig. S6A–B) and in the spatial information transfer measured through SSI (Fig. S6C–H) or MI metrics (Fig. S6I–N). However, we also noted that spatial firing profiles endowed with similar firing rate and information transfer metrics could be obtained with distinct combinations of synaptic localization profiles. Together, these results demonstrated the ability of several disparate ion-channel parametric combinations and different synaptic localization profiles to elicit similar place cell firing profiles endowed with similar information transfer profiles.

### 3.5 Regulation of spatial information transfer by experience-dependent asymmetry in place-field response profiles

Our simulations thus far resulted in symmetric place field firing profiles (*e.g.*, Fig. 4B) with a symmetric subthreshold voltage ramp (*e.g.*, Fig. 4G), consequent to the symmetric input structure defined by a Gaussian (Eq. (17)). However, electrophysiological lines of evidence from behavioral experiments point to an experience-dependent asymmetric expansion of hippocampal place fields in the direction opposite to the movement of the animal (Harvey et al., 2009; Mehta et al., 1997, 2002, 2000). What is the impact of such experience-dependent asymmetry on spatial information transfer within a single place field through place-cell rate code? To address this, we first altered the input structure to a horizontally-reflected Erlang distribution (Eq. (19)) which yielded an asymmetric place-field firing (Fig. S7A–B) profile (Seenivasan & Narayanan, 2020). Consistent with our observations with the symmetric place-field firing profile (Fig. 4), enhanced trial-to-trial variability resulted in increase in *F*_max_ (Fig. S7C) accompanied by reductions in FWHM (Fig. S7D), theta power (Fig. S7E–F) and subthreshold ramp voltage (Fig. S7G–H). The subthreshold voltage ramp profile was asymmetric (Fig. S7G), and reflected the asymmetric firing rate profile (Seenivasan & Narayanan, 2020).

**Fig. 9:**
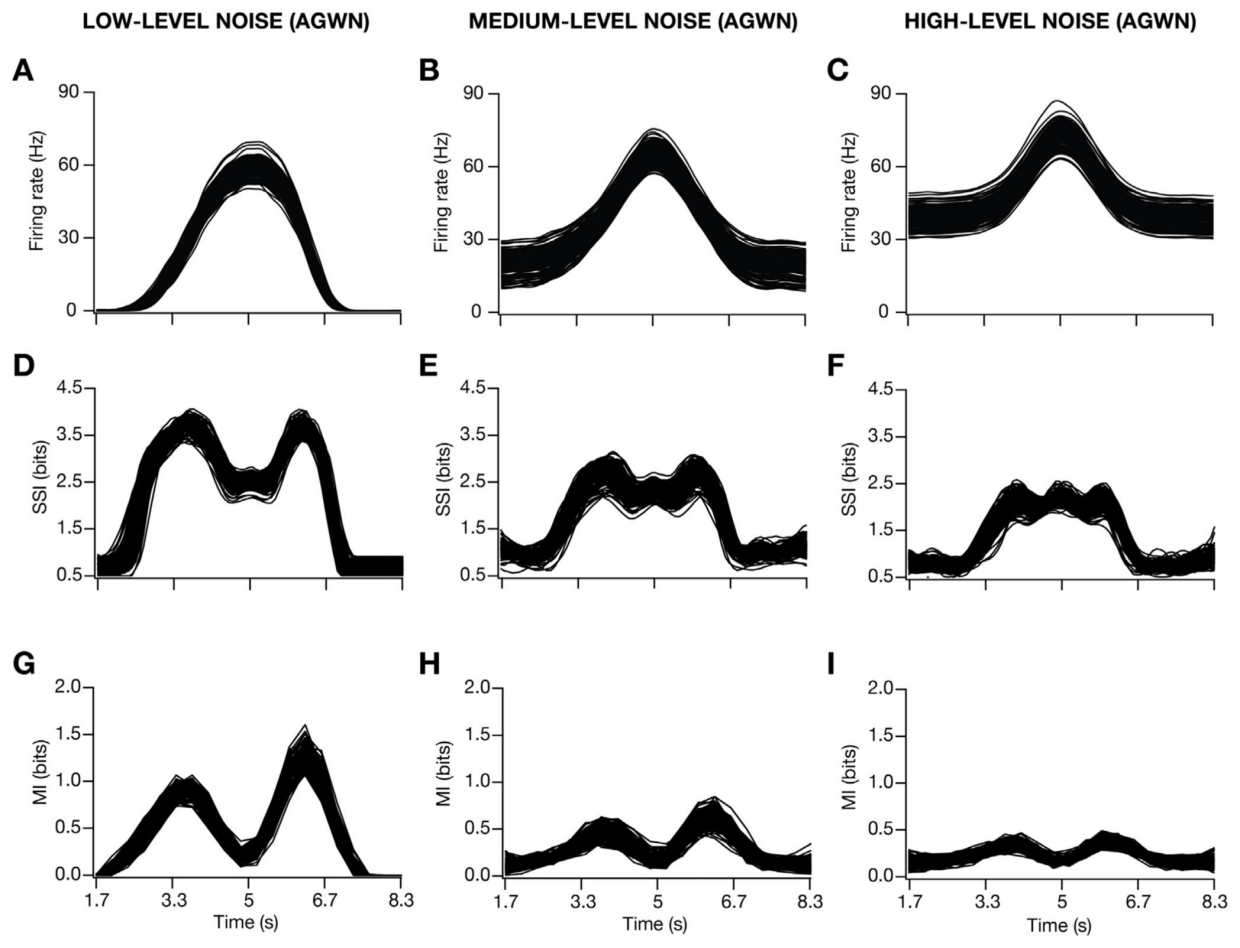
Enhanced trial-to-trial variability, imposed as an additive Gaussian white noise (AGWN), reduced spatial information transfer in models with asymmetric place-field firing. (A–I) Firing rate profiles (A–C), stimulus specific information (SSI) profiles (D–F), and mutual information profiles (G–I) as functions of time, shown for low (plots on the left), medium (plots in the middle), high (plots on the right) levels of AGWN. AGWN σ_noise_ values: *Low*: 5×10^−4^ Hz^2^, *Medium:* 1×10^−3^ Hz^2^, *High:* 5×10^−3^ Hz^2^.

**Fig. 10:**
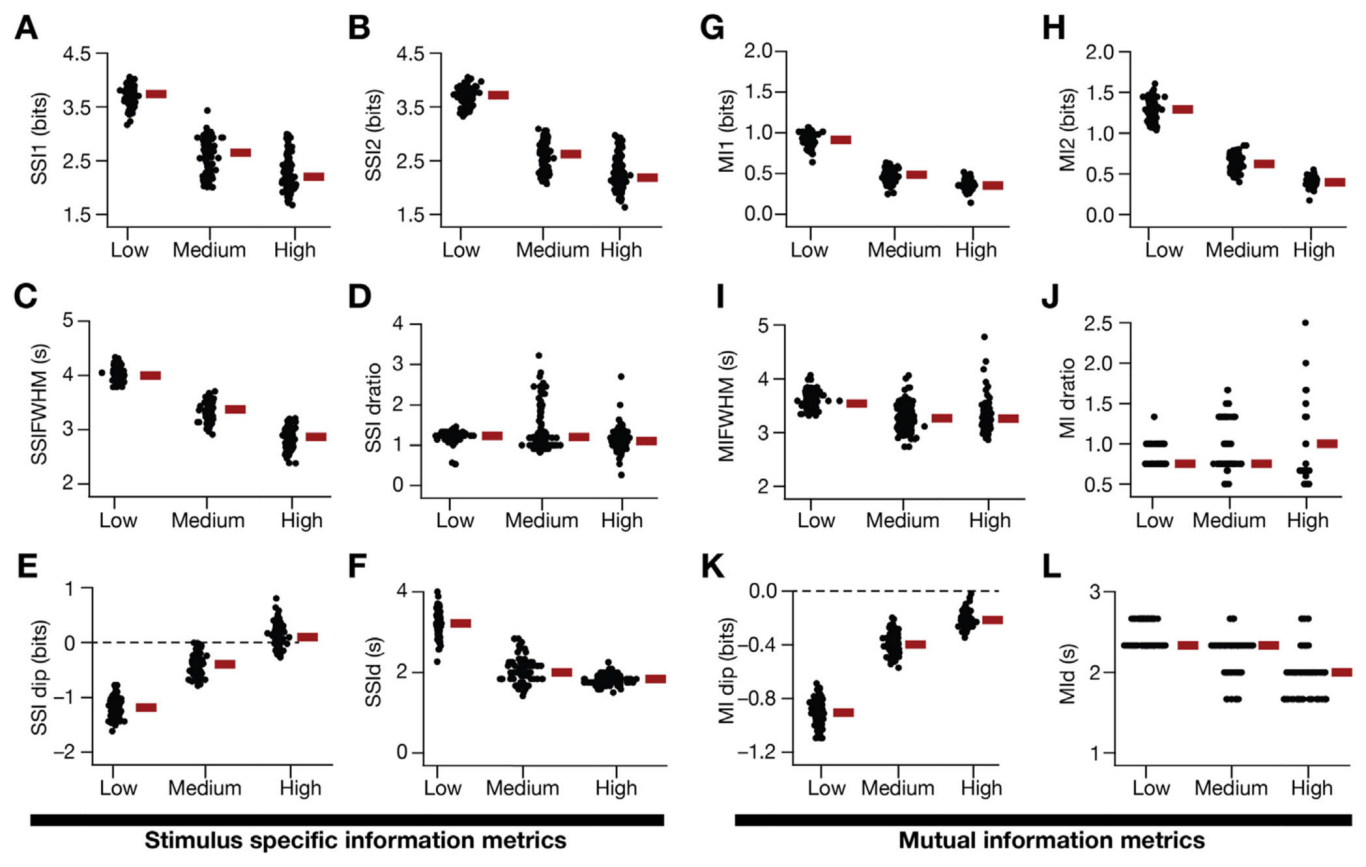
Quantification of the reduction in spatial information transfer as a consequence of enhanced trial-to-trial variability, imposed as an additive Gaussian white noise (AGWN) in models with asymmetric place-field firing. (A–F) SSI metrics for the population of valid models depicting the impact of three levels of noise on the first (A, *SSI1*) and second (B, *SSI2*) peaks of SSI, the full width half maximum of the SSI profile (C, *SSIFWHM*), the ratio of the first peak-to-center distance to the center-to-second peak distance (D, *SSI dRatio*), the difference between the SSI value at the place field center to the peak SSI value (E, *SSI dip*) and the difference between the location of *SSI1* and *SSI2* (F, *SSI d*). (G–L) Same as (A–F) for mutual information profiles of the valid model population. AGWN σnoise values: *Low*: 5×10^−4^ Hz^2^, *Medium:* 1×10^−3^ Hz^2^, *High:* 5×10^−3^ Hz^2^.

We computed the asymmetric firing rate profiles for all valid models with low (Fig. 9A), medium (Fig. 9B) and high (Fig. 9C) levels of trial-to-trial variability introduced as AGWN to the input structure (Eq. (18)). We found the baseline and the peak firing rates to shift with increased σnoise, manifesting heterogeneities across models in the populations (Fig. 9A–C). Strikingly, the stimulus-specific information transfer profiles were relatively insensitive to the asymmetry in the firing rate profile (Fig. 9D–F), although the MI profiles reflected the asymmetry (Fig. 9G–I). Specifically, the first and the second peaks were not significantly different for SSI profiles (Fig. 9D–F, Fig. 10A–B; Wilcox signed rank test between first and second peaks: Low: *p =* 0.1264; Medium: *p =* 0.1383; High: *p =* 0.2927), but the second peak was significantly larger than first peak for MI profiles (Fig. 9G–I, Fig. 10G–H; Wilcox signed rank test between first and second peaks: Low: *p =* 2.2 × 10^−16^; Medium: *p =* 2.2 × 10^−16^; High: *p =* 5.5 × 10 ^−11^) especially for low levels of trial-to-trial variability.

Consistent with our observations with a symmetric placefield profile, there was marked reduction in spatial information transfer, measured either as SSI or MI (Fig. 9D–I; Fig. 10A–B; Fig. 10G–H), with increased trial-to-trial variability. With low levels of trial-to-trial variability, we observed that the highest information transfer occurred at the high-slope regions of the firing rate profile, computed either through SSI (Fig. 10E) or MI (Fig. 10K). With increase in level of trial-to-trial variability, in a manner similar to our findings with symmetric firing profiles (Figs. 6–7) a subpopulation of models switched to transferring maximal SSI at the peak of the firing rate profile (Fig. 10E; High σ_noise_; subpopulation with *SSIdip* > 0), but no such transition occurred in the MI profile (Fig. 10K). Pairwise correlations between model physiological measurements and information metrics were mostly weak, irrespective of the level of trial-to-trial variability (Fig. S8–S10). Together, these results showed that the introduction of asymmetry in place-field firing profile introduced asymmetries in the spatial information transfer profiles computed through MI, but not through SSI.

### 3.6 The impact of activity-dependent trial-to-trial variability on spatial information transfer was minimal

**Fig. 11:**
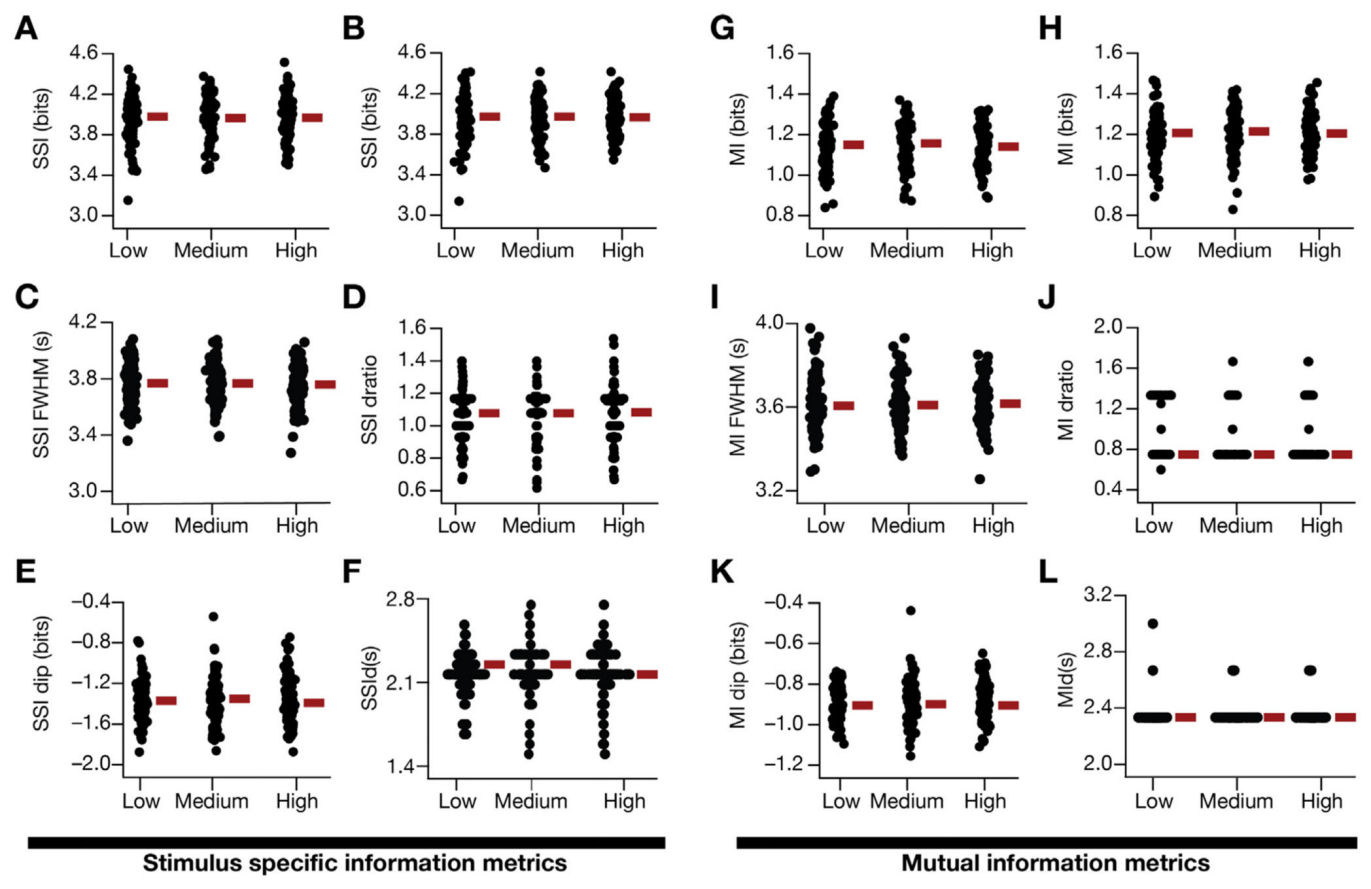
Minimal impact of enhanced activity-dependent trial-to-trial variability, imposed as a multiplicative Gaussian white noise (MGWN), on spatial information transfer. (A–F) SSI metrics for the population of valid models depicting the impact of three levels of noise on the first (B, *SSI1*) and second (C, *SSI2*) peaks of SSI, the full width half maximum of the SSI profile (D, *SSIFWHM*), the ratio of the first peak-to-center distance to the center-to-second peak distance (E, *SSI dRatio*), the difference between the SSI value at the place field center to the peak SSI value (F, *SSI dip*) and the difference between the location of *SSI1* and *SSI2* (G, *SSI d*). (G–L) Same as (A–F) for mutual information profiles of the valid model population. MGWN variance values: *Low*: 0.01 Hz^2^, *Medium*: 0.1 Hz^2^, *High*: 0.5 Hz^2^.

We had introduced trial-to-trial variability as an AGWN, whereby the variability was independent of spatial location and synaptic activity (Eq. (19)). To understand the impact of trial-to-trial variability that was dependent on synaptic activity, we introduced trial-to-trial variability as a multiplicative GWN (Eq. (20)) and repeated our analyses on spatial information transfer for the population of valid models, both with symmetric as well as asymmetric firing profiles (Fig. 11, Fig. S11–S20). Although we observed heterogeneity in firing profiles and information transfer, and found models expressing similar information transfer despite being governed by disparate parametric combinations, we found the impact of trial-to-trial variability with the higher range of σ_noise_ (compared to σ_noise_ for AGWN) to be minimal on place cell properties (Fig. S11), SSI and MI profiles (Fig. 11, Fig. S12, Figs. S16–S17) or pair-wise correlations between intrinsic and information metrics (Figs. S13–S15; Figs. S18–S20). The value of σ_noise_ employed for achieving “high” level of trial-to-trial variability (=0.5 Hz^2^) was the highest possible, as increases beyond that resulted in depolarization-induced block of action potential firing in several models. Experience-dependent asymmetry in firing profiles introduced asymmetry in the MI profiles, but not the SSI profile, even with MGWN-based trial-to-trial variability (Fig. S16–S17). In summary, our results showed that the impact of activity-dependent trial-to-trial variability is minimal compared to activity-independent variability in trial-to-trial responses, across different levels of noise and with symmetric or asymmetric place-field firing profiles.

### 3.7 Regulation of spatial information transfer by ion channel conductances and synaptic receptors

Our results established degeneracy in the emergence of place cells with similar spatial information transfer profiles, and also showed an absence of strong correlations with any physiological measurement. What contributes to such degeneracy? Are there specific ion channels that play critical regulatory roles in spatial information transfer within a place field?

**Fig. 12:**
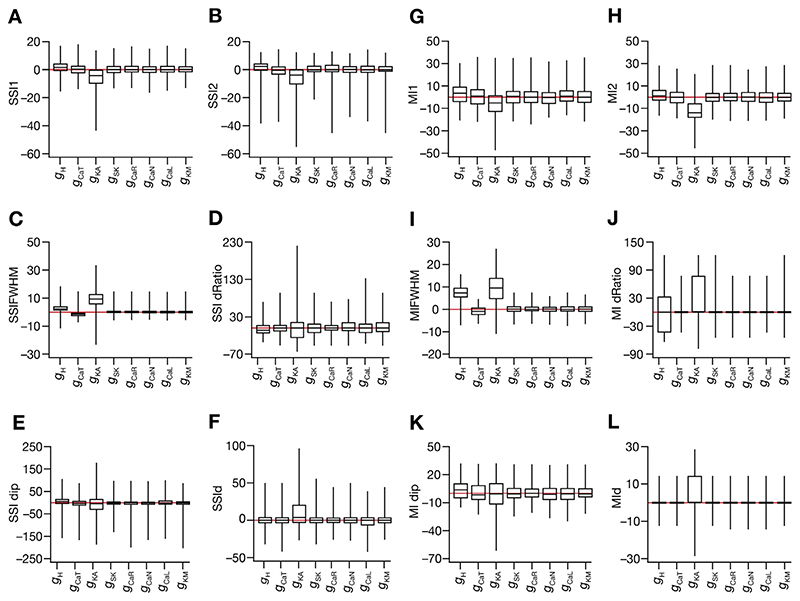
Heterogeneous impact of virtually knocking out individual ion channels on spatial information transfer in the place cell population. (A–F) Box plots showing the median and the quartiles of percentage changes in SSI-based spatial information metrics depicted in Fig. 7A as a consequence of virtually knocking out each of the 8 individual ion channels (NaF and KDR, the spike generating conductances were not knocked out because models cease spiking upon elimination of these channels). (G–L) Box plots showing the median and the quartiles of percentage changes in MI-based spatial information metrics depicted in Fig. 7H as a consequence of virtually knocking out each of the 8 individual ion channels. Plots are shown for the valid place-cell population. Red lines indicate a zero-change scenario.. (For interpretation of the references to color in this figure legend, the reader is referred to the web version of this article.)

We took advantage of our conductance-based modeling framework, and applied the virtual knockout approach (Basak & Narayanan, 2018, 2020; Jain & Narayanan, 2020; Mittal & Narayanan, 2018; Mukunda & Narayanan, 2017; Rathour & Narayanan, 2014; Seenivasan & Narayanan, 2020) to assess the contribution of individual ion channels to spatial information transfer. Specifically, we systematically assessed information transfer profiles in each of the valid models after virtually knocking out individual ion channels by setting their conductance value to zero (Fig. S21). We computed the SSI and MI metrics for the virtual knockout models (VKM) for each of the 8 active ion channels (Fig. 12). Virtual knockout of the spike generating conductances – NaF and KDR – was infeasible because the neuron ceases spiking on setting these conductance values to zero.

In terms of information transfer, we found that the impact of knocking out individual channels was heterogeneous across the model population. There were models where the SSI (Fig. 12A–B) or MI (Fig. 12G–H) values increased after knocking out the channel, but there were also models where these values decreased upon knockout. Among the channels assessed, we found the *A*-type potassium channel to have the maximal impact on spatial information transfer. Specifically, virtual knockout of the *A*-type potassium channel resulted in reductions in SSI (Fig. 12A–B) and MI (Fig. 12G–H) values (Wilcoxon signed rank *p* values: *SSI1:* 7.8×10^−9^, *SSI2*: 1.6×10^−10^, *MI1:* 2.7×10^−5^, *MI2:* 6.2×10^−15^), and increased the FWHM values of both SSI (Fig. 12C) and MI (Fig. 12I) profiles (Wilcoxon signed rank test *p* values: *SSIFWHM*: 8×10^−14^, *MIFWHM*: 2.2×10^−16^). These observations offer a clear testable prediction that *A*-type potassium channels play a critical role in regulating spatial information transfer in hippocampal place cells. These results also establish a many-to-one mapping between the different ion channels and the efficacy of spatial information transfer, whereby different ion channels could contribute towards maintaining efficacious information transfer with heterogeneous contributions across neurons in the population. This many-to-one mapping provides a substrate for the expression of degeneracy where different combinations of ion channels could maintain similar functional outcomes in terms of spatial information transfer efficacy.

**Fig. 13:**
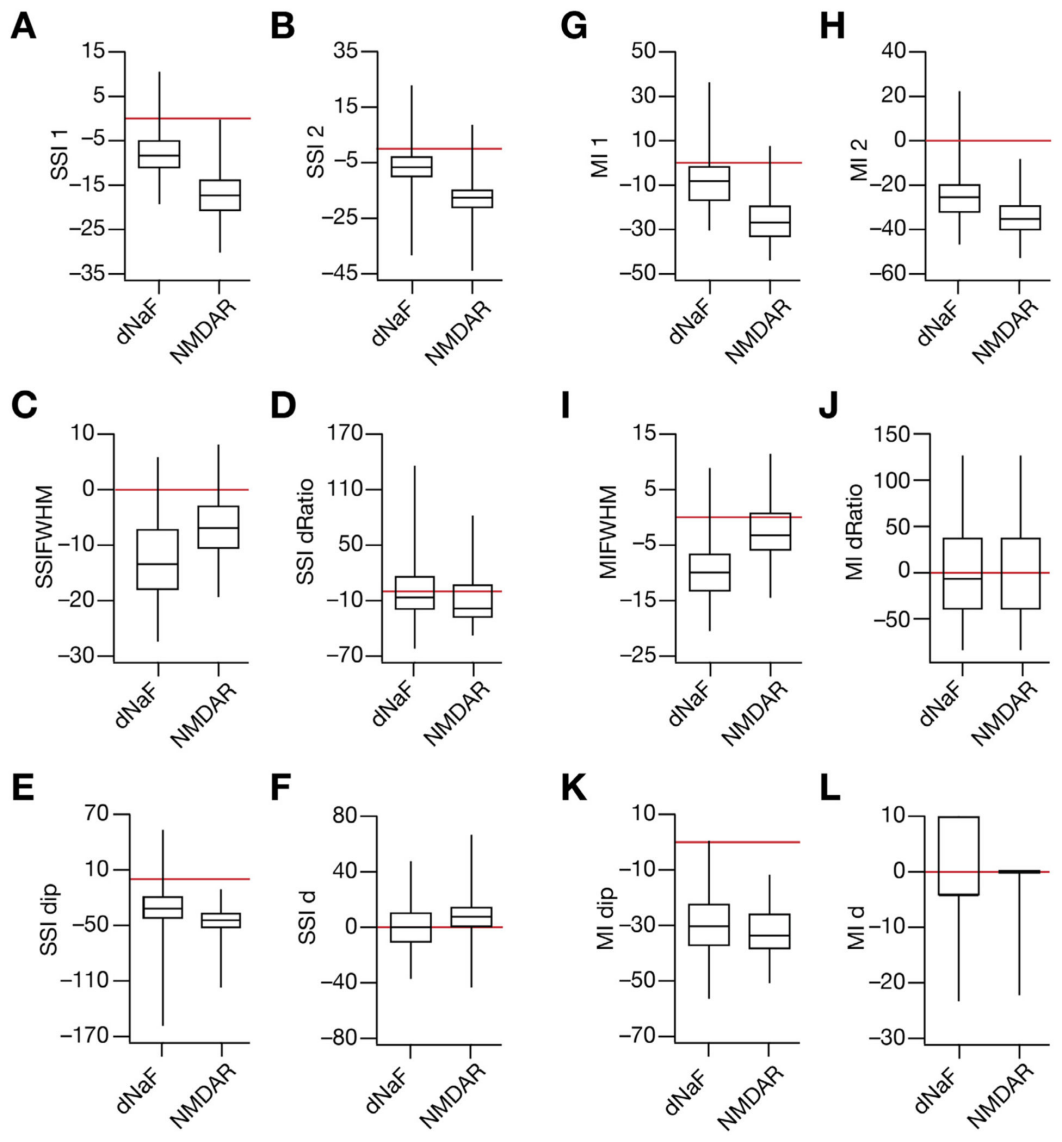
Elimination of dendritic sodium channels or NMDA receptors critically reduces spatial information transfer in the place cell population. (A–L) Box plots showing the median and the quartiles of percentage changes in SSI-based (A–F) and MI-based (G–L) spatial information metrics as a consequence of eliminating dendritic fast sodium channels (dNaF) or NMDA receptors (NMDAR). Plots are shown for the valid place-cell population. Red lines indicate a zero-change scenario.. (For interpretation of the references to color in this figure legend, the reader is referred to the web version of this article.)

Finally, as the role of NMDA receptors and dendritic spikes mediated by sodium channels expressed in the dendrites have been considered critical in place-cell physiology (Basak & Narayanan, 2018, 2020; Nakazawa, McHugh, Wilson, & Tonegawa, 2004; Sheffield, Adoff, & Dombeck, 2017; Sheffield & Dombeck, 2015), we explored the roles of these NMDARs and dendritic NaF channels in regulating spatial information transfer in our heterogeneous model population. To evaluate the role of dendritic fast sodium channels, we recomputed place-field firing rate and spatial information transfer profiles after setting the value of *ḡ_NaF_* to zero in apical dendritic compartments (Fig. S22A–B). Although there were heterogeneities in the impact of deleting dendritic sodium channels, we found a significant reduction in spatial information transfer computing either as SSI (Fig. 13A–B) or as MI (Fig. 13G–H). To assess the role of NMDARs, we recomputed place-field firing rate and spatial information transfer profiles after setting the value of *P̄*_NMDAR_ in Eqs. (9)–(11) to zero (Fig. S22C–D). Deletion of NMDARs resulted in a significant reduction in spatial information transfer (SSI: Fig. 13A–B; MI: Fig. 13G–H).

Together, these results unveiled a many-to-one relationship between the different ion channels and spatial information transfer, while also providing testable predictions on the roles of *A*-type potassium channels, NMDARs and dendritic sodium channels in regulating spatial information transfer within a single place field of hippocampal place cells.

## 4 Discussion

### 4.1 Conclusions

We demonstrated that hippocampal neurons, when they act as reliable (*i.e.*, low trial-to-trial response variability) sensors of animal location by spatially modulating their firing rate, transfer peak spatial information at the high-slope locations (and not at peak firing location) of the firing rate tuning curve within their place field. Importantly, we showed that there was significant heterogeneity across a population of models that received identical distributions of afferent synaptic patterns, owing to differences in ion channel composition of these models. The heterogeneity manifested quantitatively in terms of the amount of information transferred, and qualitatively in terms of how they responded to increases in the level of trial-to-trial variability. Specifically, with increases in trial-to-trial variability, whereas one subpopulation of models switched to transferring peak stimulus-specific spatial information at the peak-firing locations, another subpopulation continued to transfer peak information at the high-slope locations. These heterogeneities in spatial information transfer did not show strong relationships between heterogeneities in intrinsic or tuning properties of the models. We demonstrated the dependence of the spatial information transfer profile on the type of trial-to-trial variability, whereby activity-dependent variability had little impact on spatial information transfer compared to the significant reduction introduced by activity-independent variability.

To further delineate the relationship of spatial information transfer with place-cell characteristics and its components, we assessed the impact of experience-dependent asymmetry in the place-field firing rate profile. We found that mutual information metrics showed a dependence on the asymmetric nature of the firing profile, where information transfer was maximal in the second half of the place-field where the firing rate dropped at a higher rate. However, the peak values of stimulus-specific information metrics were largely invariant to the asymmetric slopes of the firing rate profile on either side of the peak-firing location. Finally, we asked if there were specific ion channels that played critical roles in regulating spatial information transfer by recomputing information metrics in models that lacked each of 8 different ion channels. We found heterogeneity in the impact of knocking out individual ion channels on these information metrics, pointing to a many-to-one relationship between different ion channel subtypes and spatial information transfer. Our analyses unveiled a potent reduction in information transfer consequent to knocking out transient potassium channels, NMDA receptors or dendritic sodium channels, providing direct experimentally testable predictions.

### 4.2 Trial-to-trial variability and spatial information transfer

Our results show that trial-to-trial variability in neural responses results in a marked reduction in spatial information transfer within a single place-field, in a manner that is dependent on how the noise was introduced. In demonstrating this, we had introduced trial-to-trial variability either an additive or a multiplicative GWN. The incorporation of synaptic additive noise is physiologically similar to a scenario where there is either a location-independent increase in afferent excitation or a reduction in tonic or spatially-uniform inhibition (Duguid, Branco, London, Chadderton, & Hausser, 2012; Grienberger et al., 2017). Such a scenario, which could be a result of physiological plasticity or pathological synaptopathies, would enhance response variability in a location-independent manner. Our results demonstrate that the presence of such location- and activity-independent enhancement in trial-to-trial variability critically reduces spatial information transfer within a place field, irrespective of whether the place field profiles are symmetric (Fig. 5, Fig. 7) or asymmetric (Figs. 8–9). With enhanced trial-to-trial variability of this form, our results show that the location of maximal SSI transitions from the high-slope regions to the peak-firing location in a subpopulation of models (Fig. 6).

In striking contrast, incorporation of trial-to-trial variability as a multiplicative noise had little impact on spatial information transfer for a wide range of noise variance values, and the location of maximal SSI was always tuned to the high-slope regions of the tuning curve (Fig. 11). Multiplicative noise, activity-dependent trial-to-trial variability, is physiologically similar to noise consequent to variability in synaptic release and receptor kinetics. In such a scenario, the amount of variability is dependent on the extent of synaptic activation, and therefore is activity-dependent. In place cells, as excitatory afferent activity is higher within the place field of the neuron (highest at the center of the place field), such multiplicative noise translates to location-dependent variability in neural responses. Our results show that the ability of such activity-dependent noise, especially with strong excitatory drives observed during place-field traversal, in altering spatial information transfer is minimal.

These results emphasize the importance of assessing the source of trial-to-trial variability and asking whether the variability is dependent or independent of activity, and caution against a generalization of all types of trial-to-trial variability to yield similar outcomes. Further explorations on the dependence of spatial information transfer on the specific types and sources of variability should account for several experimental details, some of which are listed below. First, although we consider two mutually exclusive versions of trial-to-trial variability (dependent or independent of activity), variability in neuronal responses under awake, behaving conditions is conceivably a mixture of both versions. Second, there are theoretical and electrophysiological lines of evidence for a critical role for asynchronous synaptic release, induced by active reverberation in recurrent circuits (such as the CA3, a presynaptic counterpart to the CA1 neurons studied here), on information transfer (Lau & Bi, 2005; Volman & Levine, 2009). Third, there are lines of evidence of stimulus independent noise improving the detection of subthreshold stimulus (Stacey & Durand, 2000, 2001, 2002). Fourth, although we had incorporated white noise sources in our analyses, it has been demonstrated that the color of the noise is a critical determinant of how information transfer is affected (Gingl, Kiss, & Moss, 1995). Finally, in our analyses the trial-to-trial variability was introduced solely as noise to the synaptic inputs. However, other factors such as thermal noise, noisy biochemical processes and stochasticity of ion channels could also contribute to the trial-to-trial variability, with different noise colors and different ways of interactions with the inputs (Faisal, Selen, & Wolpert, 2008; Gingl et al., 1995; Li, Luo, & Xue, 2020; Wang, Wang, & Zheng, 2014). It is essential that future studies incorporate these additional layers of mechanisms to the model and examine how different sources of variability, each with potentially different characteristics, synergistically affect stimulus-specific information content. It is possible that one or the other version dominates under specific physiological/pathological conditions, and therefore it is important that the variability-inducing mechanisms are delineated before the impact of such variability is assessed.

### 4.3 Place-cell characteristics and spatial information transfer

An important insight obtained from our study pertains to parametric degeneracy in effectuating spatial information transfer in place cells, with reference to ion channels and parameters that govern place cell biophysics and physiology (Figs. S1–S2; Fig. 3). Ion-channel degeneracy in the hippocampal formation is ubiquitous, and expresses across different scales of analyses (Mishra & Narayanan, 2019, 2021; Mittal & Narayanan, 2018; Rathour & Narayanan, 2019). In hippocampal CA1 pyramidal neurons, the expression of degeneracy has been demonstrated with reference to the concomitant emergence of several somatodendritic intrinsic properties (Migliore et al., 2018; Rathour et al., 2016; Rathour & Narayanan, 2012, 2014; Srikanth & Narayanan, 2015), spike-triggered average (Das & Narayanan, 2014, 2015, 2017, 2017; Jain & Narayanan, 2020), short- (Mukunda & Narayanan, 2017) as well as long-term (Anirudhan & Narayanan, 2015) plasticity profiles. Degeneracy has been shown to express in the sharpness of place-field firing properties with reference to biophysical as well as morphological parameters (Basak & Narayanan, 2018, 2020), which has been confirmed in this study with a larger set of ion channels incorporated into the model. Finally, an earlier study had quantitatively defined efficiency of phase coding in hippocampal place cells and showed that similar spatial information transfer could be achieved with disparate ion channel combinations (Seenivasan & Narayanan, 2020). The findings of this study, demonstrating ion channel degeneracy with reference to spatial information transfer through the rate code within a single place field, further strengthen the expression of degeneracy in encoding systems such as the hippocampus (Rathour & Narayanan, 2019).

In encoding systems, it is essential that encoding of information occurs concurrently with maintenance of homeostasis of intrinsic neuronal properties, including neuronal firing rate (Rathour & Narayanan, 2019). In our study, we showed that similar amounts of spatial information transfer and similar firing rate (both with reference to place-field firing and responses to pulse currents) could concomitantly occur with disparate combinations of ion channel conductances and parameters that govern their expression (Table S1, Fig. S2). It has been shown that the balance between excitation, inhibition and intrinsic excitability (E–I–IE balance) is essential for achieving concomitant efficient phase coding as well as activity homeostasis. In our study, we had fixed the excitatory synaptic weights to account for synaptic democracy (Fig. 1I) and did not incorporate spatially-uniform inhibition (Grienberger et al., 2017) as this would have translated to merely a negative bias term across locations (Basak & Narayanan, 2018). We also found that there were no correlations between information measurements and other intrinsic measurements (*e.g.*, Figs. S3–S5). Future studies could alter excitatory synaptic weights associated with place-field inputs and explore the balance between excitation, location-dependent inhibition and the heterogeneous intrinsic excitability properties of hippocampal pyramidal neurons to assess the role of E–I–IE in the emergence of efficient information transfer through rate codes as well. Specifically, such studies could validate models based on their ability to transfer maximal spatial information through the rate code (*i.e.*, efficient rate coding) *and* concomitantly maintain intrinsic homeostasis, and ask if E–I–IE was essential to achieve these when the search space involves excitatory/inhibitory synaptic weights and ion channel conductances (Seenivasan & Narayanan, 2020). Importantly, such models could maximize the *joint* spatial information transfer occurring through the rate as well as the phase codes (Mehta et al., 2002; O’Keefe & Burgess, 2005) within a place field, and explore the constraints required for such efficient encoding to occur simultaneously with the expression of intrinsic homeostasis.

Degeneracy in the emergence of similar spatial information transfer and signature intrinsic properties emerged as a consequence of a many-to-one relationship between ion channels and spatial information transfer. These observations were feasible only because we employed a heterogeneous population of models, derived from an unbiased stochastic search that covered heterogeneities in the underlying parameters (Marder & Taylor, 2011). If we had instead resorted to the use of a single hand-tuned model to arrive at our conclusions, that single model and its specific composition would have biased our results. In such a scenario, the identification of the aforementioned many-to-one relationship and the consequent heterogeneities on the impact of individual ion channels on information transfer would not have been feasible. These results emphasize the critical role of synergistic interactions among different ion channels in effectuating behavior, and underscore that the impact of any ion channel subtype is dependent on the relative expression profiles of other channels and receptors in the specific model under consideration.

Degenerate systems show dominance of specific underlying parameters in regulating *specific* physiological measurements (Basak & Narayanan, 2018, 2020; Drion, O’Leary, & Marder, 2015; Mishra & Narayanan, 2019; Mittal & Narayanan, 2018; Mukunda & Narayanan, 2017; Rathour et al., 2016; Rathour & Narayanan, 2014, 2019). In our analyses, although we found that all ion channels had the ability to reduce or increase spatial information transfer in a model-dependent manner (Figs. 12–13, certain parameters played a crucial role in regulating information transfer. Specifically, our analyses provide specific experimentally testable predictions on the critical roles of dendritic sodium channels, NMDA receptors and *A*-type potassium channels in regulating spatial information transfer (Figs. 12–13). Interestingly, these three components play critical roles in regulating the prevalence of dendritic spikes and in the sharpness of place-cell tuning profiles (Basak & Narayanan, 2018, 2020; Gasparini, Migliore, & Magee, 2004; Golding, Jung, Mickus, & Spruston, 1999; Golding & Spruston, 1998; Losonczy & Magee, 2006), and form strong candidates in regulating spatial information transfer. Further studies could test the roles of these channels in regulating information transfer in hippocampal pyramidal neurons employing electrophysiological recordings during place-field traversal in the presence of pharmacological agents. As these components alter dendritic spiking in opposite directions (suppressing NMDA receptors or sodium channels suppresses dendritic spiking, whereas suppression of *A*-type potassium channels enhances dendritic spiking), such studies could also potentially assess the requirement of an intricate balance between mechanisms that promote and those that prevent dendritic spike initiation in maintaining efficient spatial information transfer.

Our results proffer a testable prediction that experience-dependent asymmetry in place-field profiles do not markedly alter SSI. As experience-dependent asymmetry is considered to be predictive, reduction in spatial information transfer during the early parts of place-field firing would have rendered this predictive capability to be ineffectual. Our observations demonstrate that although the low values of slope during the early parts of firing profile reduces mutual information as a consequence of the asymmetry, stimulus specific information remains high. Further explorations could test this prediction on electro-physiologically obtained individual place cells transitioning with experience (Mehta et al., 1997).

With specific reference to hippocampal place fields, future studies could explore the impact of systematic gradients in neuronal properties and ion channel expression along the dorsoventral, proximo-distal and superficial-deep axes of the hippocampus (Cembrowski et al., 2016; Cembrowski & Spruston, 2019; Danielson et al., 2016; Dougherty, Islam, & Johnston, 2012; Dougherty et al., 2013; Kjelstrup et al., 2008; Lee et al., 2014; Malik et al., 2016; Marcelin et al., 2012; Maroso et al., 2016; Mizuseki, Diba, Pastalkova, & Buzsaki, 2011; Strange et al., 2014; Sun et al., 2017) on spatial information transfer within a single place field. In this context, our analyses assumed a fixed constant velocity and had focused on the impact of biophysical parameters from a *relative* perspective, with reference to fixed place-field widths (defined by *F_pre_*(*t*)). Future analyses of spatial information transfer should relax these assumptions, and account for gradients in place-field width along the dorso-ventral axis (Kjelstrup et al., 2008; Strange et al., 2014), along with speed-dependence of hippocampal network physiology (Buzsaki, 2002; Colgin, 2016; McFarland, Teitelbaum, & Hedges, 1975; McNaughton, Barnes, & O’Keefe, 1983; Sławińska & Kasicki, 1998).

Finally, the question on how spatial information transfer is regulated by activity-dependent plasticity and behavioral state-dependent neuromodulation of ion channels and receptors is critical in understanding the emergence of spatial information transfer in the context of novel place-field formation (Basak & Narayanan, 2018; Bittner et al., 2015, 2017; Cohen, Bolstad, & Lee, 2017; Kim & Lim, 2020; McKenzie et al., 2021; Robinson et al., 2020; Sheffield et al., 2017; Zhao, Wang, Spruston, & Magee, 2020). Future studies should therefore assess the impact of novel spatial environments, place-cell remapping, and different forms of neural plasticity on spatial information transfer. In this context, as with many other studies on the neurophysiology of place cells and their formation (Ahmed & Mehta, 2009; Basak & Narayanan, 2018, 2020; Bittner et al., 2015, 2017; Dombeck et al., 2010; Dragoi & Buzsaki, 2006; Geisler et al., 2010; Grienberger et al., 2017; Harvey et al., 2009; Huxter et al., 2003; Lee et al., 2012; Mehta et al., 1997, 2002, 2000; Seenivasan & Narayanan, 2020), our study analyzes animal traversal in a one-dimensional arena. Although one-dimensional arenas have proven to be useful approximations and have provided several important insights about place cell physiology and plasticity, it is critical to recognize that external space is not one-dimensional. There are emergent features of place cells in two and three dimensions that are not captured by one-dimensional arenas (Aghajan et al., 2015; Finkelstein, Las, & Ulanovsky, 2016; Geva-Sagiv, Las, Yovel, & Ulanovsky, 2015; Huxter, Senior, Allen, & Csicsvari, 2008; Lee, Briguglio, Cohen, Romani, & Lee, 2020; Moser et al., 2017, 2015; Rich, Liaw, & Lee, 2014; Wang, Xu, & Wang, 2018; Yartsev & Ulanovsky, 2013). As animals interact with the real world, from an ethological perspective, it is essential that analyses on the impact of neural heterogeneities and trial-to-trial variability on spatial information transfer are expanded to two- and three-dimensional place field inputs. Future studies should therefore extend our conductance-based morphologically realistic analysis of the cellular neurophysiology of spatial information transfer to two- as well as three-dimensional virtual arenas.

From a broader perspective, our analyses here focused only on the relationship between spatial information transfer and spatially modulated neuronal firing rate. However, the hippocampal formation has been implicated in other functions, such as recognition, completion and separation of patterns, associative memory, and in engram formation (Andersen et al., 2006; Josselyn & Tonegawa, 2020). Future studies should therefore focus on the possibility that there could be other molecular and cellular constraints that define the hippocampal architecture towards satisfying these additional functions, apart from accounting for energy considerations associated with neuronal and network physiology (Attwell & Laughlin, 2001; Laughlin, 2001; Laughlin, de Ruyter van Steveninck, & Anderson, 1998; Wang, Wang, & Zhu, 2017; Wang, Xu, & Wang, 2019; Zhu, Wang, & Zhu, 2018).

## Supplementary Material

Supplementary material related to this article can be found online at https://doi.org/10.1016/j.neunet.2021.07.026.

Supplementary Fig. S1 to Supplementary Fig. S22 & Supplementary Table S1

## Figures and Tables

**Table 1:** Model parameters, their base values and ranges for stochastic search. For all parameters, the range uniformly spanned 0.5–2× of the respective base model value.

	Parameter (unit)	Symbol	Base value	Range
Passive properties				
*R*_a_ (uniform across the neuron)				
1	Axial resistivity (Ω-cm)	*R_a_*	120	100–250
*R*_m_ (sigmoidal reduction with distance from soma)				
2	Maximum value (kΩ cm ^−2^)	$R_{m}^{\text{soma}\;}$	125	62.5–250
3	Minimum value (kΩ cm^−2^)	$R_{m}^{\text{end}\;}$	85	42.5–170
4	Half-maximal point of sigmoid (μm)	$R_{m}^{\text{hmp}\;}$	300	150–600
5	Slope of sigmoid (μm)	$R_{m}^{\text{slope}\;}$	50	25–100
Active properties				
Spike-generating channels (uniform across all somatodendritic compartments)				
6	Maximum conductance for NaF (mS cm^−2^)	${\bar{g}}_{Na}$	16	8–32
7	Maximum conductance for KDR (mS cm^−2^)	${\bar{g}}_{KDR}$	10	5–20
HCN channel (sigmoidal increase with distance from soma)				
8	Maximum somatic conductance (μS cm^−2^)	${\bar{g}}_{h}{\;}^{\text{soma}\;}$	25	12.5–50
9	Fold increase	${\bar{g}}_{h}{\;}^{\text{fold}}$	12	6–24
10	Half-maximal point of sigmoid (μm)	${\bar{g}}_{h}{\;}^{\text{hmp}}$	320	160–640
11	Slope of sigmoid (μm)	${\bar{g}}_{h}{\;}^{\text{slope}}$	50	25–100
*T*-type calcium channel (sigmoidal increase with distance from soma)				
12	Maximum somatic conductance (μS cm^−2^)	${\bar{g}}_{CaT}^{\text{soma}}$	80	40–160
13	Fold increase	${\bar{g}}_{CaT}^{\text{fold}}$	30	15–60
14	Half-maximal point of sigmoid (μm)	${\bar{g}}_{CaT}^{\text{hmp}}$	350	175–700
15	Slope of sigmoid (μm)	${\bar{g}}_{CaT}^{\text{slope}}$	50	25–100
*A*-type potassium channel (linear increase with distance from soma)				
16	Maximum somatic conductance (mS cm^−2^)	${\bar{g}}_{KA}^{\text{soma}}$	3.1	1.55–6.2
17	Fold increase per 100 μm	${\bar{g}}_{KA}^{\text{fold}}$	8	4–16
*N*-type calcium channel (till 340 μm from soma in apical dendrites)				
18	Maximum conductance	${\bar{g}}_{CaN}$	15	7.5–30
*R*-type calcium channel (dendritic localization)				
19	Maximum conductance in dendrites (μS cm^−2^)	${\bar{g}}_{\text{CaR}}$	15	7.5–30
*L*-type calcium channel (perisomatic, till 50 μm from soma in apical dendrites)				
20	Maximum conductance (mS cm^−2^)	${\bar{g}}_{CaL}$	1.20	0.6–2.4
Small-conductance calcium-activated potassium channel (dendritic localization)				
21	Maximum conductance of SK (μS cm^−2^)	${\bar{g}}_{SK}$	1.5	0.75–3
*M*-type potassium channel (perisomatic, till 50 μm from soma)				
22	Maximum conductance (μS cm^−2^)	${\bar{g}}_{KM}$	1	0.5–2

**Table 2:** Intrinsic somatodendritic measurements of CA1 pyramidal neurons and their electrophysiological bounds for validating models. Bounds on intrinsic somatodendritic functional maps and firing rate measurements were derived from electrophysiological recordings reported in Malik, Dougherty, Parikh, Byrne, and Johnston (2016), Narayanan et al. (2010), Narayanan and Johnston (2007, 2008) and Spruston et al. (1995). Bounds on place-cell tuning sharpness are relative in nature, where cells with high firing rate and low FWHM were selected (Basak & Narayanan, 2018, 2020).

	Measurement	Soma		~150 μm		~300 μm	
		Lower	Upper	Lower	Upper	Lower	Upper
Intrinsic somatodendritic functional map measurements (18)							
1	Input Resistance (MΩ)	40	100	30	60	10	50
2	Maximum Impedance (MΩ)	50	110	35	80	20	80
3	Resonance frequency (Hz)	2	7	4	8	5	14
4	Strength of Resonance	1.01	1.5	1.01	1.9	1.2	2.6
5	Total Inductive Phase (rad Hz)	0	0.3	0	1	0.025	2
6	Backpropagating Action Potential (mV)	90	115	40	70	5	45
Action potential firing rate measurements (4)							
7	Firing rate for 100 pA current injection	0	20				
8	Firing rate for 150 pA current injection	0	30				
9	Firing rate for 200 pA current injection	0	40				
10	Firing rate for 250 pA current injection	5	45				
Measurements of place-cell tuning sharpness (2)							
11	Peak firing rate (Hz)	56	–				
12	Full Width at Half Maxima (s)	–	2.5				

**Table 3:** Quantitative metrics of information transfer.

Measurement name	Symbol
SSI-based information metrics (Fig. 7A)	
1st peak of the SSI curve	SSI1
2nd peak of the SSI curve	SSI2
Full width at half maximum of the SSI curve	SSI FWHM
Ratio of the distance between middle peak with 1st peak and the distance	SSI dRatio
between middle peak and 2nd peak of the SSI curve	
SSI middle peak value - average of SSI peak values at the slopes	SSI dip
Temporal distance between the two peaks in the SSI curve	SSId
MI-based information metrics (Fig. 7H)	
1st peak of the MI curve	MI1
2nd peak of the MI curve	MI2
Full width at half maximum of the MI curve	MI FWHM
Ratio of the distance between middle peak with 1st peak and the distance	MI dRatio
between middle peak and 2nd peak of MI curve	
MI middle peak value – average of MI peak values at the slopes	MI dip
Temporal distance between the two peaks in MI curve	Mid
